# Fibrinography and thrombography (thrombodynamics-4D) in atrial fibrillation assessment of direct oral anticoagulants in geriatrics patients aged 80 years and older receiving direct oral anticoagulant therapy

**DOI:** 10.1016/j.rpth.2025.102969

**Published:** 2025-07-15

**Authors:** Geoffrey Foulon-Pinto, Georges Jourdi, Maxime Delrue, Carmelo Lafuente-Lafuente, Candice Cavalie, Isabelle Gouin-Thibault, Julien Le Guen, Pascale Gaussem, Tristan Mirault, Etienne Puymirat, Thomas Lecompte, Eric Pautas, Emmanuel Curis, Virginie Siguret

**Affiliations:** 1 INSERM UMR_S1140, Université Paris Cité, Paris, France; 2 Hématologie Biologique, Hôpital Lariboisière, Assistance Publique-Hôpitaux de Paris, Paris, France; 3 INSERM UMR_S1144, Université Paris Cité, Paris, France; 4 GH Pitié Salpêtrière-Charles Foix–Gériatrie à orientation cardiologique et neurologique, Sorbonne Université, Ivry-sur-Seine, France; 5 Clinical Epidemiology and Ageing Team, INSERM, IMRB, Université Paris-Est Créteil, Créteil, France; 6 Service d’Hématologie Biologique, CHU de Rennes, Rennes, France; 7 INSERM CIC 1414 (Centre d’Investigation Clinique de Rennes), CHU de Rennes, Université de Rennes, Rennes, France; 8 Gériatrie aiguë, Hôpital Européen Georges Pompidou, Assistance Publique-Hôpitaux de Paris, Paris, France; 9 Hématologie Biologique, Hôpital Européen Georges Pompidou, Assistance Publique-Hôpitaux de Paris, Paris, France; 10 Médecine vasculaire, Hôpital Européen Georges Pompidou, Assistance Publique-Hôpitaux de Paris, INSERM U970 PARCC team 5, Université Paris Cité, Paris, France; 11 Cardiologie, Hôpital Européen Georges Pompidou, Assistance Publique-Hôpitaux de Paris, Paris, France; 12 Médecine vasculaire, CHRU Nancy, Université de Lorraine, Nancy, France; 13 GH Pitié Salpêtrière-Charles Foix–Gériatrie aiguë polyvalente, Sorbonne Université, Ivry-sur-Seine, France; 14 UR 7537 BioSTM, Faculté de Pharmacie, Université Paris Cité, Paris, France

**Keywords:** atrial fibrillation, direct oral anticoagulant, elderly, fibrin formation, pharmacodynamics, thrombin generation

## Abstract

**Background:**

Scarce data are available on pharmacodynamic (PD) variability in very elderly patients receiving direct oral anticoagulants (DOACs) for atrial fibrillation (AF). Thrombodynamics-4D (TD-4D), which simultaneously assesses fibrin clot formation and thrombin generation, has not yet been tested in patients on rivaroxaban, apixaban, or dabigatran.

**Objectives:**

To (i) evaluate TD-4D’s ability to assess DOAC effect added to normal plasma; (ii) assess DOAC PD in very elderly patients with AF along with DOAC concentrations; (iii) identify factors associated with interindividual variability of DOAC PD at peak and trough levels.

**Methods:**

Assessment of Direct oral Anticoagulants in Geriatrics (NCT02464488) is a prospective, multicenter study including inpatients aged ≥80 years receiving DOACs for AF for at least 4 days. Fibrinography and thrombography parameters were measured using TD-4D along with plasma DOAC concentrations (antifactor [F]Xa or anti-FIIa activity) and fibrinogen.

**Results:**

We analyzed pooled normal plasma samples spiked with DOACs and 345 samples from 187 Assessment of Direct oral Anticoagulants in Geriatrics patients (mean ± SD, age 87 ± 4 years; 69% females): 69 on rivaroxaban, 70 on apixaban, and 48 on dabigatran. All 3 DOACs prolonged fibrinography lag time and decreased initial rate of clot growth and clot size at 30 minutes in a concentration-dependent manner in spiking experiments and patients. DOACs prolonged temporal thrombography parameters while decreasing thrombin peak height and endogenous thrombin potential. At trough, apixaban and dabigatran concentrations were the only significant predictors of interindividual variability in both thrombin peak height (thrombography) and initial rate of clot growth (fibrinography). In rivaroxaban patients, cardiac failure significantly influenced thrombin peak height variability.

**Conclusion:**

Fibrinography and thrombography, assessed simultaneously with TD-4D, provided consistent results for all 3 DOACs, including dabigatran. Substantial PD variability was observed, partly influenced by DOAC concentrations. The clinical relevance of such variability remains to be demonstrated.

## Essentials

• Data on PD variability in very elderly patients on DOACs are scarce.
• We assessed thrombography/fibrinography (Thrombodynamics) in patients ≥80 years old, along with DOAC levels.
• DOACs, including dabigatran, exerted a significant concentration-dependent effect on most thrombography/fibrinography parameters.
• Substantial PD variability was observed, partly influenced by DOAC concentrations.

## 1 Introduction

The prevalence of nonvalvular atrial fibrillation (AF) increases with aging, reaching >10% in patients aged ≥80 years [1,2]. Particular attention must be paid to very elderly patients with AF as they frequently have numerous comorbid conditions and are polymedicated, leading to high risk for both thrombosis and bleeding. Current guidelines recommend the use of direct oral anticoagulants (DOACs), ie, inhibitors of factor (F)Xa (rivaroxaban, apixaban, and edoxaban) or thrombin (dabigatran), as first-line treatment in the prevention of systemic embolic events in patients with AF [3,4]. In the last decade, meta-analyses of randomized clinical trials and real-life studies have confirmed the favorable clinical benefit-risk ratio of these drugs compared with vitamin K antagonists in this fragile population [[5], [6], [7], [8]]. Given their large therapeutic margin and predictable pharmacokinetics (PK) and pharmacodynamics (PD), DOACs can be administered at fixed dose without laboratory monitoring, except in specific situations. However, PK studies have documented substantial interindividual variability in DOAC plasma concentrations, regardless of the regimen (full or reduced dose), which was greater in octogenarians and nonagenarians than that observed in younger patients [[9], [10], [11], [12], [13], [14], [15], [16], [17], [18]]. In the Assessment of Direct oral Anticoagulants in Geriatrics (ADAGE) study conducted by our group in patients aged ≥80 years with AF receiving rivaroxaban or apixaban, we evidenced important (>50%) coefficients of variation (CVs) in both maximal (Cmax) and minimal plasma concentrations of both anti-FXa inhibitors [17]. Regarding the assessment of DOAC PD, the study of thrombin generation (TG) has been shown to be a suitable and reliable assay to assess *in vitro* or *ex vivo* anticoagulant effect of direct FXa inhibitors, in contrast to prothrombin time [17,[19], [20], [21], [22], [23], [24], [25], [26], [27], [28], [29], [30], [31]]. In ADAGE study, substantial variability in thrombin peak heights was noticed, especially at low concentration intervals for both rivaroxaban and apixaban, suggesting an impact of the underlying coagulation status of elderly inpatients [17]. While thrombography has been widely evaluated in patients on DOACs in different settings, the effect of DOACs on fibrinography, ie, fibrin clot formation and growth (taking into account not only fibrinogen level), and the potential relationships between thrombography and fibrinography have been poorly investigated. It was therefore of particular interest to further evaluate DOAC PD in ADAGE patients, in whom hyperfibrinogenemia is common, using the Thrombodynamics-4D (TD-4D) analyzer. Indeed, this unique system has the advantage of enabling complete phenotyping of both TG and fibrin clot growth [32,33].

To date, TD-4D has already been shown to be suitable for exploring hypercoagulability states in different settings [[34], [35], [36], [37], [38], [39]], as well as hypocoagulability states. It is particularly used in clinical practice to monitor patients receiving heparin therapy in some countries [[40], [41], [42], [43]]. However, very few studies with a limited number of patients (<10 for each DOAC) have assessed DOAC PD using TD-4D [44].

In the present study, we first evaluated the ability of TD-4D to assess the effect of DOACs (rivaroxaban, apixaban, and dabigatran) in spiked pooled normal plasma samples. Then, in plasma samples from ADAGE patients, we sought to (i) characterize the PD profile of DOACs using TD-4D, along with DOAC concentrations; (ii) identify factors associated with interindividual variability in DOAC PD parameters at peak and trough concentrations.

## 2 Methods

### 2.1 Materials

#### 2.1.1 Pooled normal plasma samples spiked with rivaroxaban, apixaban, or dabigatran

Frozen pooled normal plasma samples (Cryocheck-Cryopep) were spiked with increasing concentrations of DOACs (0-450 ng/mL), as previously described [45]. Rivaroxaban, apixaban, and dabigatran were kindly provided by Bristol Myers Squibb/Pfizer, Bayer Healthcare, and Boehringer Ingelheim, respectively.

#### 2.1.2 ADAGE patients and sampling

ADAGE is an academic, prospective, observational multicenter study (NCT 02464488; for details, see Foulon-Pinto et al. [17]). We recruited hospitalized patients from the university hospital’s geriatric department (Assistance Publique-Hôpitaux de Paris, Paris, France). Briefly, patients were eligible if they were aged ≥80 years and received rivaroxaban, apixaban, or dabigatran for AF for at least 4 days. The DOAC dose regimen was at the discretion of the physician. Exclusion criteria included any acute, unstable comorbid condition or an estimated life expectancy of a few weeks. Sampling (see below) and last DOAC intake times were recorded on a standardized request form. Sampling time points were at the discretion of the physicians over a period of 20 days following inclusion. Time points were classified according to the time elapsed from the last oral intake (T, hours) for patients receiving rivaroxaban. T1 to T4 corresponded to peak time (Tmax), and T20 to T24 corresponded to trough (Tmin). For patients receiving apixaban or dabigatran (twice daily), T1 and T4 corresponded to Tmax, and T10 to T12 corresponded to Tmin. Physicians prospectively collected demographic, clinical, and laboratory data (including creatinine clearance [CrCl] calculated with the Cockcroft–Gault formula). The study was approved by the institutionnel Ethics Committee (Comité consultatif de protection des personnes pour la recherche biomédicale–Île-de-France-6–number 15.074bis). All participants gave written informed consent to participate in the study. Only patients for whom remaining plasma aliquots were available were included in the present study.

### 2.2 Methods

#### 2.2.1 Blood collection and processing

Blood samples in patients and controls were collected by venepuncture into a citrate tube (buffered trisodium citrate 0.109 M, 9:1 v/v). Tubes were sent to each centre’s laboratory within 2 hours after sampling and immediately double-centrifuged for 15 minutes at 2000 × *g* at 15 to 22 °C to obtain platelet-poor plasma. Plasma samples (500 μL aliquots) were stored at −80 °C prior to shipment on dry ice for central analysis (Haematology Laboratory, Hôpital Lariboisière). Plasma samples were thawed for 3 to 5 minutes in a 37 °C water bath just before testing.

#### 2.2.2 Measurement of plasma DOAC and fibrinogen levels

We measured DOAC concentrations (expressed in nanograms per milliliter [ng/mL]) in plasma samples with the STA-R analyzer (Stago) using chromogenic anti-FXa assays (STA-Liquid Anti-FXa, Stago) for rivaroxaban and apixaban, and anti-FIIa activity using Hemoclot Direct Thrombin Inhibitors (Hyphen-Biomed) for dabigatran. Dedicated calibrations and controls were used. The lower limit of quantification was 20 ng/mL for the 3 assays.

Plasma fibrinogen levels were centrally measured in each sample using Clauss method (Dade Thrombin Reagent, Siemens).

#### 2.2.3 TD-4D assay

The TD-4D assay was performed centrally using the Thrombodynamics analyzer system (Hemacore, Diapharma) with the TD-4D PLS dedicated kit (Hemacore, Diapharma). The system simultaneously measures spatiotemporal parameters of fibrin clot formation (fibrinography) and TG process (thrombography) in a thin layer of unstirred plasma [32,41,42,46]. Briefly, 120 μL of platelet-poor plasma is added to a microcuvette containing corn trypsin inhibitor to prevent contribution of contact phase activation, then 5 μL of phospholipid solution (manufacturer’s reagent) is added. After incubation for 15 minutes at 37 °C in the Thrombodynamics device, the mixture is transferred to a second microcuvette containing calcium ions, and then immediately transferred to the measurement cuvette. Finally, the activator insert, whose surface is coated with immobilized tissue factor (100 pmol/m^2^), is then transferred to the cuvette. The process of fibrin clot formation is recorded in a time-lapse video-microscopy mode using a dark-field light scattering method. Simultaneously, the system monitors TG by fluorimetry, based on the ability of thrombin to cleave a fluorogenic substrate, 7-amino-4-methylcoumarin (AMC) bound to a short amino acid sequence (Arg-Gly-Gly). The rate of AMC formation is proportional to local thrombin concentration. The microcuvette is maintained at 37 °C and is illuminated with red (625 nm) or ultra-violet (365 nm) light emitting diodes. Clot growth is detected with red light scattering, while AMC fluorescence is detected with UV. Scattered red light and fluorescence passing through the macro lens are recorded by a charge-coupled device system. Images under red and blue light are acquired once per minute. The whole analysis lasts 1 hour. The Thrombodynamics software (Hemacore, Diapharma) calculates parameters of spatiotemporal dynamics of fibrin clot formation and TG [40,46].

The following fibrinography parameters were recorded (see Supplementary Table 1): (i) lag time (Tlag, minutes); (ii) initial rate of clot growth (Vi, μm/min); (iii) rate of clot growth (V, μm/min); (iv) clot size (CS, μm) measured at 30 minutes; (v) clot density (D, arbitrary units); (vi) time to spontaneous clotting (Tsp, minutes). The following thrombography parameters (at the near-activator area) were recorded (see Supplementary Table 1): (i) Tlag (minutes); (ii) time to thrombin peak (minutes); (iii) maximal thrombin concentration (ie, thrombin peak height, Cmax_ATG, AU/L); (iv) endogenous thrombin potential (ETP_ATG, AU⋅min/L); (v) stationary amplitude of moving peak of thrombin (Ast, AU/L); (vi) rate of thrombin peak propagation (μm/min; see Supplementary Videos 1–3).

#### 2.2.4 TD-4D assay performances—reference intervals

Interassay CVs for all parameters measured in pooled normal plasma (Cryocheck) were <8.0%, except for Ast and Tsp (Supplementary Table 2). Mean values of fibrinography and thrombography parameters were within the manufacturer’s reference intervals [35,40], except for D. The lower D we observed is thought to result from the additives present in Cryocheck, leading to hypodense clots. We locally tested frozen plasma samples from 30 DOAC-free volunteers, with a mean (SD) age of 31 ± 8 years, enrolled in the Dabigatran etexilate and Rivaroxaban Influence of Genetics factors in healthy volunteers (DRIVING) study [25] (Supplementary Table 2). Reference intervals were established as mean ± 2 SD and were of the same order of magnitude as those of the manufacturer [40].

In addition, we tested samples from randomly selected 35 elderly subjects, referred to the Haematology Laboratory of Lariboisière University Hospital (Paris, France) for coagulation testing, aged ≥80 years, without any anticoagulant treatment, and having normal full blood count and routine coagulation assay results (including prothrombin time ratio, activated partial thromboplastin time, and fibrinogen level). In accordance with French regulations, they all approved the use of their leftover plasma samples via a nonopposition form. Their mean (SD) age was 85 ± 5 years (minimum-maximum [min-max], 80-95 years), with 66% being females; half had mild to normal renal function, and half had moderate renal failure. Their mean fibrinography and thrombography parameters were within the manufacturer’s reference intervals, except for Vi and CS, which were slightly higher [35,40] (Supplementary Table 2).

#### 2.2.5 Statistical analysis

Quantitative variables were expressed as mean ± SD, CV, median (IQR [25th-75th percentiles]), or min-max. The associations between DOAC concentrations, fibrinography, and thrombography parameters were tested using Spearman’s rank correlation. Comparisons between ADAGE patients and DOAC-free elderly subjects were performed using Mann–Whitney *U*-test for each DOAC.

We prespecified that the Vi (fibrinography) and Cmax_ATG (thrombography) would be chosen as the 2 Thrombodynamics parameters for statistical analysis, given their sensitivity to a wide range of DOAC concentrations. Potential predictors of Vi (fibrinography) and Cmax_ATG (thrombography), including intake of amiodarone, any CYP450 inhibitor, any P-glycoprotein inhibitor or substrate, CrCl, and the presence of heart failure, at Tmin and Tmax, were tested using either Mann–Whitney *U*-test (categorical predictor) or Spearman tests (quantitative predictor, univariate analysis). To account for confounding effects of DOAC or fibrinogen concentrations (for Vi) on the parameters, multivariate analysis was also performed using a linear model with both predictors and confounders. The *P* value for the predictor was computed using type I sums of squares, with the predictor being the last term in the model.

Statistical analyses were conducted using R (version 4.0.2) [47] and Python (version 3.9.12) [48], with the latter in a Jupyter Notebook environment (version 6.4.8). Python libraries, including pandas [49], NumPy [50], matplotlib [51], seaborn [52], and SciPy [53], were used for additional data processing and analysis. A *P* value < .05 was considered statistically significant after multiplicity correction; all results are given as raw *P* values.

## 3 Results

### 3.1 TD-4D results in normal plasma spiked with DOACs

**Figure 1:**
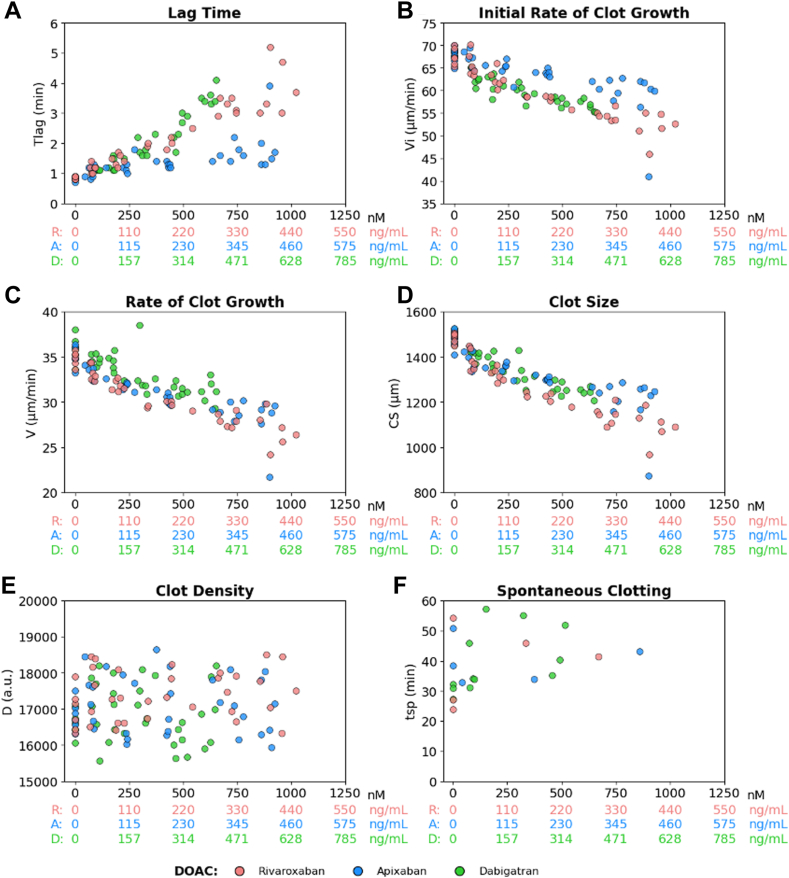
Fibrinography parameters (Thrombodynamics-4D) as a function of direct oral anticoagulant (DOAC) concentrations in spiking experiments. (A) Lag time (Tlag), (B) initial rate of clot growth (Vi), (C) rate of clot growth (V), (D) clot size (CS) at 30 minutes, (E) clot density (D), and (F) time to spontaneous clotting (Tsp). Pooled normal plasma (Cryocheck) samples were spiked with rivaroxaban (R), apixaban (A), or dabigatran (D) to obtain final concentrations ranging from 0 to 1000 nM. DOAC concentrations were measured in the spiked samples. Concentrations are expressed in both nanograms per milliliter (ng/mL) and nanomolar (nM) to facilitate comparisons between R (red dots), A (blue dots), and D (green dots). Spontaneous clotting occurred in *n* = 5 (14%), *n* = 5 (14%), and *n* = 12 (33%) samples spiked with R, A, and D, respectively, mainly at low DOAC concentrations.

We first evaluated fibrinography parameters using TD-4D as a function of DOAC concentrations, using pooled normal plasma samples spiked with rivaroxaban, apixaban, or dabigatran (Figure 1). The 3 DOACs prolonged Tlag and decreased rate of growth and CS at 30 minutes in a concentration-dependent manner (*P* < 10^-4^). Early Tsp (<30 minutes) occurred only in 2.8% of the samples.

**Figure 2:**
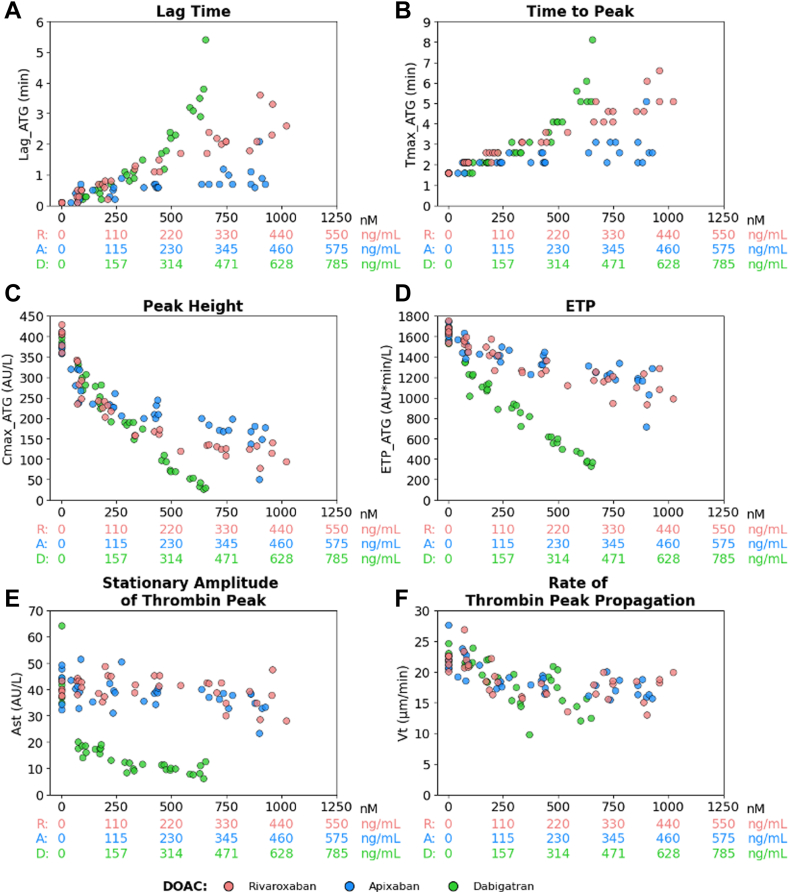
Thrombography parameters (Thrombodynamics) as a function of direct oral anticoagulant (DOAC) concentrations in spiking experiments. (A) Lag time (Lag_ATG), (B) time to peak (Tmax_ATG), (C) maximal thrombin concentration (peak height, Cmax_ATG), (D) endogenous thrombin potential (ETP_ATG), (E) stationary amplitude of thrombin moving peak (Ast), and (F) rate of thrombin peak propagation (Vt). Pooled normal plasma (Cryocheck) samples were spiked with rivaroxaban (R), apixaban (A), or dabigatran (D; see Figure 1 legend). Concentrations of DOACs are expressed in both nanograms per milliliter (ng/mL) and nanomolar (nM) to facilitate comparisons between R (red dots), A (blue dots), and D (green dots). ETP, endogenous thrombin potential.

Regarding thrombography, increasing DOAC plasma concentrations were significantly associated with prolonged temporal parameters (Lag_ATG and Tmax_ATG), while Cmax_ATG, ETP_ATG, and rate of thrombin peak propagation decreased (*P* < 10^−4^) with each DOAC (Figure 2). Ast also decreased with increasing concentrations of apixaban (*P* = .0039) and dabigatran (*P* < 10^-4^), but not rivaroxaban (*P* = .269; Figure 2). Dabigatran had a more pronounced effect on Cmax_ATG, ETP_ATG, and Ast than rivaroxaban or apixaban (Figure 2).

### 3.2 DOAC PD in ADAGE patient samples using TD-4D

#### 3.2.1 Patient characteristics

A total of 345 plasma samples from 187 ADAGE patients were analyzed with TD-4D. Patient characteristics are summarized in Table 1. The mean (SD) age was 87 ± 4 years (min-max, 80-98 years), with 69% of the patients being female. Sixty-nine patients received rivaroxaban, 70 received apixaban, and 48 received dabigatran; most patients received a reduced dose (Table 1).

**Table 1:** Characteristics of the Assessment of Direct oral Anticoagulants in Geriatrics patients with Thrombodynmics-4D results at inclusion.

Patient characteristics	Rivaroxaban	Apixaban	Dabigatran	Whole cohort
	*n* = 69	*n* = 70	*n* = 48	*N* = 187
**Demographic characteristics**				
Age (y), mean ± SD	86.4 ± 4.2	87.0 ± 4.1	86.1 ± 4.3	86.6 ± 4.2
Females, %	69.6	72.9	62.5	69.0
Body weight (kg), mean ± SD	65.2 ± 13.0	67.4 ± 16.1	67.0 ± 16.0	66.5 ± 14.9
**Clinical characteristics**				
CHA_2_DS_2_VASc score, mean ± SD	4.9 ± 1.4	5.1 ± 1.3	4.2 ± 1.6	4.9 ± 1.4
HEMORR_2_HAGES score, mean ± SD	2.0 ± 1.0	2.6 ± 1.1	2.1 ± 0.8	2.3 ± 1.0
CIRS-G score, mean ± SD	9.5 ± 3.9	11.7 ± 5.2	9.1 ± 3.4	10.4 ± 4.6
Previous stroke or TIA, %	26.5	30.4	27.7	28.3
Diabetes mellitus, %	10.6	27.5	20.0	19.4
Heart failure, %	54.4	53.6	23.4	46.2
**Laboratory data, mean** ± **SD**				
CrCl^a^ (mL/min)	50.8 ± 18.0	49.2 ± 15.5	54.5 ± 22.1	50.9 ± 18.1
Albumin (g/L)	33.1 ± 3.9	32.9 ± 6.1	32.3 ± 5.9	32.9 ± 5.2
Hemoglobin (g/dL)	12.5 ± 1.4	11.7 ± 1.8	12.4 ± 1.5	12.2 ± 1.6
Fibrinogen (g/L)	5.0 ± 1.8	5.2 ± 1.3	4.4 ± 1.7	4.9 ± 1.6
**Therapeutic data, %**				
Reduced dose DOAC^b^	88.4	77.1	100.0	87.2
No. of comedications, mean ± SD	6.1 ± 2.7	6.6 ± 2.9	5.4 ± 2.2	6.1 ± 2.7
≥1 CYP3A4/5 inhibitor	14.9	27.5	22.7	21.7
≥1 P-glycoprotein inhibitor	37.3	47.8	43.2	42.8
Amiodarone	14.9	27.5	18.2	20.6
Antiplatelet therapy	10.6	24.6	2.3	14.0

CHA_2_DS_2_VASc, congestive heart failure, hypertension, age ≥75 (doubled), diabetes mellitus, prior stroke or transient ischemic attack (doubled), vascular disease, age 65–74, female; CIRS-G, Cumulative Illness Rating Scale-Geriatrics; CrCl, creatinine clearance; DOAC, direct oral anticoagulant; HEMORR_2_HAGES, hepatic or renal disease, ethanol abuse, malignancy, older age, reduced platelet count or function, re-bleeding, hypertension, anemia, genetic factors, excessive fall risk and stroke; TIA, transient ischemic attack.

a Calculated using Cockcroft–Gault formula.

b Rivaroxaban 15 mg once daily, apixaban 2.5 mg twice daily, and dabigatran 110 mg twice daily.

The average Cumulative Illness Rating Scale-Geriatrics score of 10.5 showed substantial comorbid conditions. The mean CrCl (Cockcroft–Gault) was 51.0 mL/min. Polymedication was frequent, with an average of 6.1 drugs per patient in addition to DOACs (Table 1).

Of the 345 samples, 148 were collected from patients on rivaroxaban (with an average of 2.1 time points per patient), 129 from patients on apixaban (1.8 time points per patient), and 68 from patients on dabigatran (1.4 time points per patient).

#### 3.2.2 Association of DOAC concentrations with fibrinography parameters in ADAGE patients

**Figure 3:**
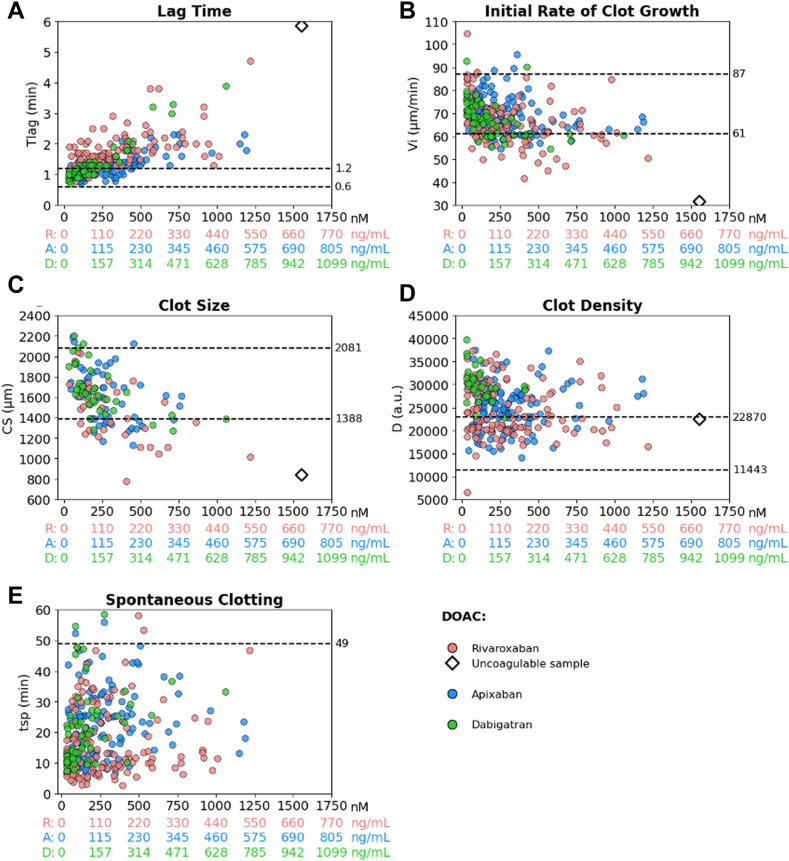
Fibrinography parameters (Thrombodynamics) as a function of direct oral anticoagulant (DOAC) concentrations in all Assessment of Direct oral Anticoagulants in Geriatrics (ADAGE) patients. (A) Lag time (Tlag), (B) initial rate of clot growth (V), (C) clot size (CS) at 30 minutes, (D) clot density (D), and (E) time to spontaneous clotting (Tsp). Concentrations of DOACs are expressed in both nanograms per milliliter (ng/mL) and nanomolar (nM) to facilitate comparisons between rivaroxaban (R; red dots), apixaban (A; blue dots), and dabigatran (D; green dots). Dashed lines indicate the locally determined reference intervals in healthy subjects. Of note, 1 patient on R (676 ng/mL) is represented by an open black diamond (beyond the limits of quantification). a.u., arbitrary units.

In ADAGE patients, DOAC plasma concentrations ranged from < 20 ng/mL to 676 ng/mL, 547 ng/mL, and 665 ng/mL for rivaroxaban, apixaban, and dabigatran, respectively. Tlag was prolonged, and the Vi measured in the 2- to 6-minute interval decreased with increasing DOAC concentration (*P* < 10^−4^), irrespective of the DOAC (Figure 3). The CS decreased with increasing rivaroxaban, apixaban, and dabigatran concentrations (*P* = .0002, *P* = .002, *P* < 10^−4^, respectively). Tlag, Vi, and CS values presented substantial variability in the 3 DOACs at a given concentration interval (eg, 100-200 ng/mL; Figure 3).

D was significantly associated with dabigatran concentration (*P* = .0048), but not with rivaroxaban or apixaban concentrations. D was strongly associated with plasma fibrinogen level of the 3 DOACs (*P* < 10^-4^ for rivaroxaban and apixaban; *P* = .032 for dabigatran; Supplementary Figure 1). Remarkably, Tsp occurred in 94% of ADAGE patient samples, with an early median time of 18 minutes (IQR, 10-27), which hampered the recording of data for V (measured within the 15-25-minute interval) and CS (measured at 30 minutes) in a substantial number of samples. There was no relationship between time to spontaneous clot formation and rivaroxaban or apixaban levels, but a significant association with dabigatran levels *(P* = .0001; Figure 3).

#### 3.2.3 Association of DOAC concentrations with thrombography parameters in ADAGE patients

**Figure 4:**
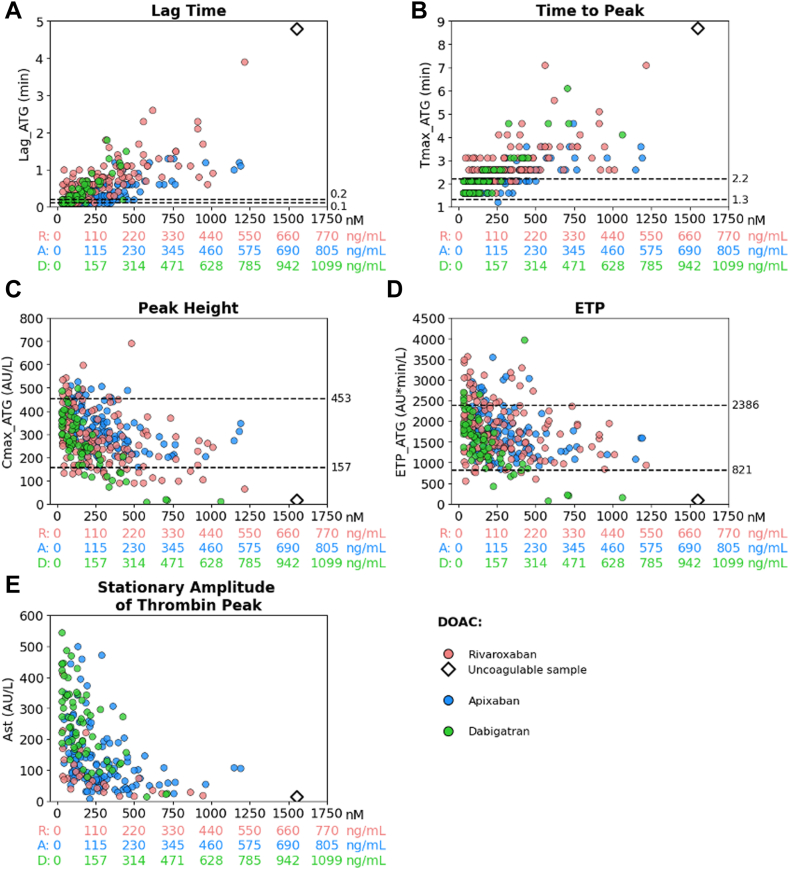
Thrombography parameters (Thrombodynamics-4D) as a function of direct oral anticoagulant (DOAC) concentrations in all Assessment of Direct oral Anticoagulants in Geriatrics (ADAGE) patients. (A) Lag time (Lag_ATG), (B) time to peak (Tmax_ATG), (C) maximal thrombin concentration (Cmax_ATG), (D) endogenous thrombin potential (ETP_ATG), and (E) stationary amplitude of thrombin moving peak (Ast). Concentrations of DOACs are expressed in both nanograms per milliliter (ng/mL) and nanomolar (nM) to facilitate comparisons between rivaroxaban (R; red dots), apixaban (A; blue dots), and dabigatran (D; green dots). Dashed lines indicate the locally determined reference intervals in healthy subjects. Of note, 1 patient on R (676 ng/mL) is represented by an open black diamond (beyond the limits of quantification). AU, arbitrary units; ETP, endogenous thrombin potential.

Tlag and time to peak were prolonged with increasing DOAC plasma concentrations (*P* < 10^−4^), while Cmax_ATG (*P* < 10^−4^) and ETP_ATG decreased (*P* = .0009, *P* = .0001, *P* < 10^−4^ for rivaroxaban, apixaban, dabigatran, respectively; Figure 4). Cmax_ATG and ETP_ATG values presented marked interindividual variability in the 3 DOACs (Figure 4).

#### 3.2.4 Relationships between thrombography and fibrinography parameters

Strong associations (*P* < 10^-4^ for most parameters) were found between thrombography and fibrinography parameters, especially for temporal parameters in ADAGE patients (Supplementary Figure 2) as well as in spiked samples (not shown). These results demonstrate that the delay in clot formation and the decrease in clot growth reflect the decrease in TG induced by DOACs.

#### 3.2.5 PK and PD variability in ADAGE patients at Tmax and Tmin

**Figure 5:**
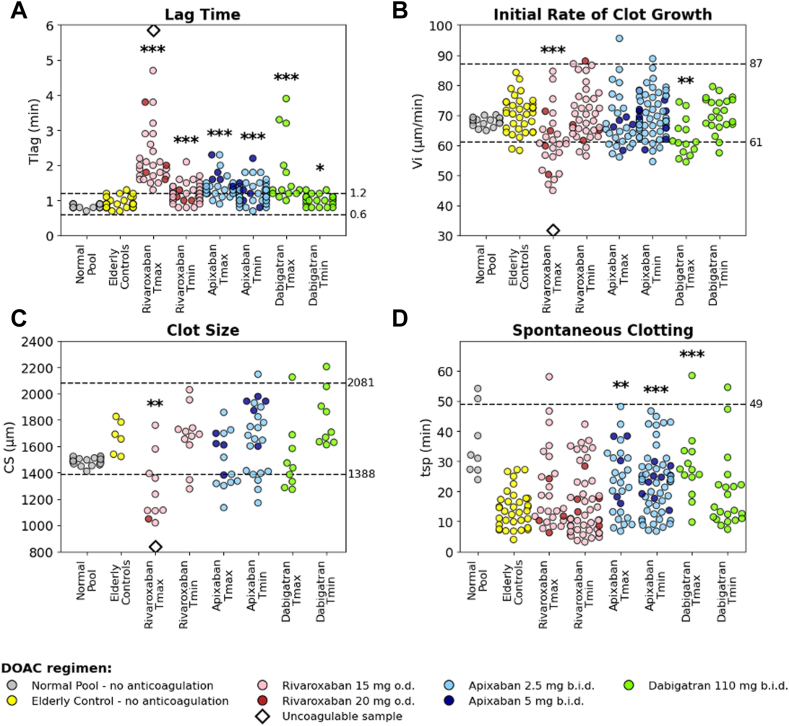
Fibrinography parameters (Thrombodynamics-4D) in a subset of Assessment of Direct oral Anticoagulants in Geriatrics (ADAGE) patients on rivaroxaban, apixaban, or dabigatran (reduced dose) at peak time (Tmax) and trough (Tmin) compared with pooled normal plasma (Normal Pool; gray) and elderly direct oral anticoagulant (DOAC)-free subjects (Elderly control; yellow). (A) Lag time (Tlag), (B) initial rate of clot growth (Vi), (C) clot size (CS) at 30 minutes, and (D) time of spontaneous clotting (Tsp). Fibrinography parameters are presented as scattered dot plots. ADAGE patients on rivaroxaban o.d. 15 mg (pink) or 20 mg (red); ADAGE patients on apixaban twice daily (b.i.d.) 2.5 mg (light blue) or 5.0 mg (dark blue); ADAGE patients on 110 mg dabigatran twice daily (green). Dashed lines indicate the locally determined reference intervals in healthy subjects. Comparisons between the mean values of each parameter in the Elderly control group and ADAGE patients are shown as ∗∗∗*P* < 10^-3^, ∗∗*P* < 10^-2^, ∗*P* < .05 (Mann–Whitney U-test). Of note, 1 patient on rivaroxaban (676 ng/mL) is represented by an open black diamond (beyond the limits of quantification). b.i.d., twice-daily; o.d., once daily.

**Figure 6:**
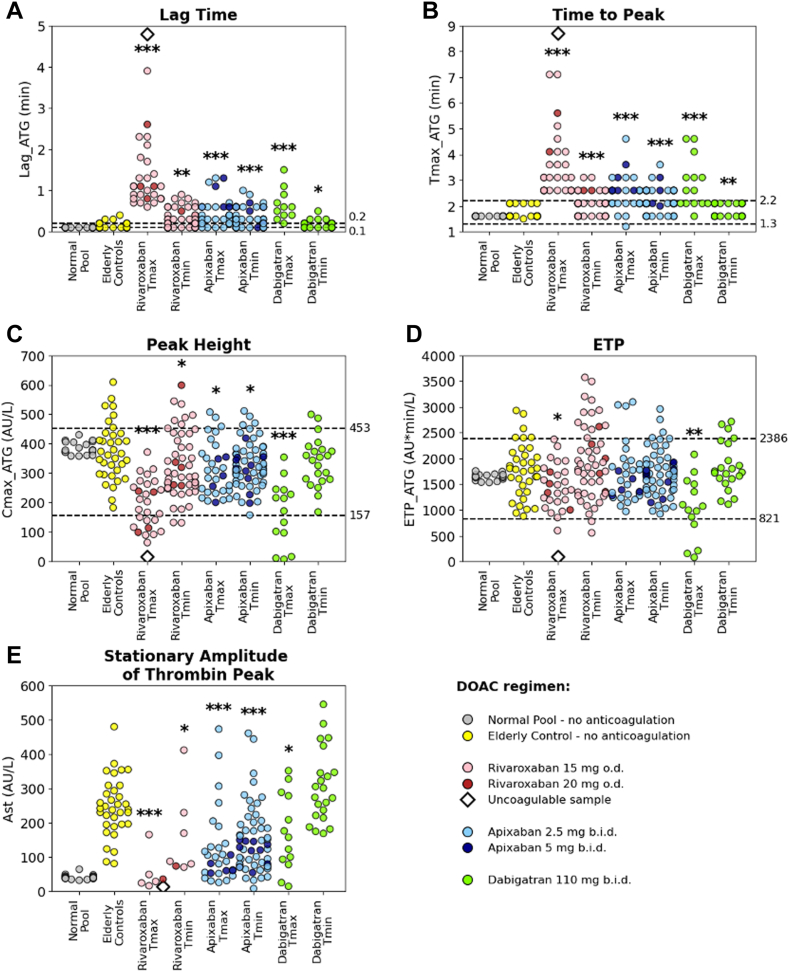
Thrombography parameters in a subset of Assessment of Direct oral Anticoagulants in Geriatrics (ADAGE) patients on rivaroxaban, apixaban, or dabigatran (reduced dose) at peak time (Tmax) and time to trough (Tmin) compared with pooled normal plasma (Normal Pool; gray) and direct oral anticoagulant (DOAC)-free elderly subjects (Elderly control; yellow). (A) Lag time (Lag_ATG), (B) time to peak (Tmax_ATG), (C) maximal thrombin concentration (Cmax_ATG), (D) endogenous thrombin potential (ETP_ATG), and (E) stationary amplitude of thrombin moving peak (Ast). Thrombography parameters are presented as scattered dot plots. ADAGE patients on rivaroxaban o.d. 15 mg (pink) or 20 mg (red); ADAGE patients on apixaban 2.5 mg (light blue) or 5.0 mg (dark blue) twice daily; ADAGE patients on 110 mg dabigatran (green) twice daily. Dashed lines indicate the locally determined reference intervals in healthy subjects. Comparisons between the mean values of each parameter in the elderly control group and ADAGE patients are shown as ∗∗∗*P* < 10^-3^, ∗∗*P* < 10^-2^, ∗*P* < .05 (Mann–Whitney *U*-test). Of note, 1 patient on rivaroxaban (676 ng/mL) is represented by an open black diamond (beyond the limits of quantification). AU, arbitrary units; b.i.d., twice-daily; ETP, endogenous thrombin potential; o.d., once daily.

We then focused on the subset of ADAGE patients who had a time point at Tmax or Tmin (Tables 2 and 3). In the subset of patients receiving a reduced dose, ie, 15 mg o.d. for rivaroxaban, 2.5 mg twice daily for apixaban, and 110 mg twice daily for dabigatran, we observed a wide interindividual variability in DOAC concentrations at both Tmax and Tmin, with CVs varying from 47% to 67% (Table 2). In contrast, regarding PD, CVs were <30% for fibrinography parameters, regardless of the DOAC, except for Tlag at Tmax in rivaroxaban (CV = 36%) and dabigatran (CV = 51%), and for time to Tsp of the 3 DOACs (Figure 5). Regarding thrombography, a greater variability was observed in Tlag (CV > 50%) of the 3 DOACs. For Cmax_ATG and ETP_ATG, the variability was remarkably low in patients receiving apixaban (CVs < 34%) at both time points (Table 3). The highest variability was observed in Cmax_ATG at Tmax in patients receiving rivaroxaban or dabigatran (CV = 41% and 63%, respectively), and at Tmax for ETP_ATG in patients receiving dabigatran (CV = 56%; Figure 6).

**Table 2:** Direct oral anticoagulant concentrations and fibrinography parameters (Thrombodynmics-4D) at peak time (Tmax) and trough (Tmin) in the Assessment of Direct oral Anticoagulants in Geriatrics patients with reduced direct oral anticoagulant dose.

Parameter	Rivaroxaban 15 mg once daily		Apixaban 2.5 mg twice daily		Dabigatran 110 mg twice daily	
	Tmax	Tmin	Tmax	Tmin	Tmax	Tmin
**DOAC concentration (ng/mL)**						
Samples, *n*	24	43	25	49	14	22
Median (IQR)	248 (181-397)	42 (27-69)	170 (133-210)	74 (49-117)	171 (123-280)	39 (20-70)
Range	64-676	20-149	69-442	20-240	79-665	20-106
CV, %	49.5	66.0	46.8	59.5	66.7	58.9
**Tlag (min)**						
Samples, *n*	22	32	24	47	14	22
Median (IQR)	2.0 (1.7-2.6)	1.2 (1.0-1.4)	1.3 (1.2-1.4)	1.1 (1.0-1.3)	1.3 (1.2-2.0)	1.0 (0.9-1.1)
Range	1.3-4.7	0.8-2.1	0.9-2.3	0.7-1.8	1.0-3.9	0.8-1.3
CV, %	36.0	23.0	21.9	21.6	51.0	15.3
**Initial rate of clot growth (μm/min)**						
Samples, *n*	22	32	24	47	14	22
Median (IQR)	60.6 (56.2-65.3)	70.4 (64.9-75.8)	64.9 (60.9-74.2)	68.3 (64.8-76.2)	60.9 (57.8-65.6)	70.3 (66.9-75.1)
Range	45.0-84.6	56.5-87.1	56.1- 95.5	54.6-88.8	54.5-74.4	57.5-79.5
CV, %	16.4	11.5	14.2	10.5	9.6	7.6
**CS (μm)**						
Samples, *n*	9	11	10	17	9	9
Median (IQR)	1236 (1112-1393)	1691 (1632-1734)	1362 (1317-1698)	1655 (1395-1780)	1436 (1332-1583)	1707 (1634-1904)
Range	1019-1759	1276-2029	1134-1857	1170-2147	1273-2125	1612-2205
CV, %	18.0	12.5	15.0	15.6	16.8	11.1
**D (a.u.)**						
Samples, *n*	23	32	24	47	5	13
Median (IQR)	23,230 (20,060-28,420)	23,940 (20,550-30,160)	25,340 (22,600-27,790)	25,470 (21,190-29,910)	28,810 (27,570-29,170)	32,220 (30,160-36,290)
Range	14,690-33,710	18,160-37,370	14,090-34,880	15,530-37,550	22,740-29,360	26,190-39,810
CV (%)	21.1	21.8	19.0	22.1	9.0	11.4
**Time to Tsp (min)**						
Samples, *n*	21	42	23	44	13	21
Median (IQR)	18.5 (11.4-31.6)	10.9 (7.1-23.1)	22.9 (12.1-31.1)	23.0 (12.8-28.1)	27-2 (24.7-33.2)	13.7 (11.0-22.2)
Range	7.7-58.1	3.2-42.3	6.8-48.2	6.8-46.7	9.8-58.5	7.4- 54.6
CV, %	62.9	69.7	49.8	49.2	39.7	63.0

a.u., arbitrary units; CS, clot size; CV, coefficient of variation; D, clot density; DOAC, direct oral anticoagulant; Tlag, lag time; Tmax, peak time; Tmin, trough; Tsp, spontaneous clotting.

**Table 3:** Thrombography parameters (Thrombodynmics-4D) at peak time and trough in the Assessment of Direct oral Anticoagulants in Geriatrics patients with reduced direct oral anticoagulant dose.

Parameter	Rivaroxaban 15 mg once daily		Apixaban 2.5 mg twice daily		Dabigatran 110 mg twice daily	
	Tmax	Tmin	Tmax	Tmin	Tmax	Tmin
**Tlag (min)**						
Samples, *n*	23	43	25	49	11	22
Median (IQR)	1.1 (0.8-1.5)	0.2 (0.1-0.4)	0.3 (0.2-0.6)	0.2 (0.2-0.6)	0.6 (0.4-0.8)	0.2 (0.1-0.2)
Range	0.6-3.9	0.1- 0.9	0.1-1.3	0.1-1.0	0.2-1.5	0.1-0.5
CV, %	56.9	79.5	71.0	69.9	55.8	54.1
**Time to peak (min)**						
Samples, *n*	23	43	25	49	14	22
Median (IQR)	3.1 (2.6-3.8)	2.1 (1.6-2.1)	2.1 (2.1-2.6)	2.1 (2.1-2.1)	2.6 (2.1-3.1)	1.85 (1.6-2.1)
Range	2.6-7.1	1.6-3.1	1.2-4.6	1.6-3.6	1.6-4.6	1.6-2.1
CV, %	35.8	18.4	26.9	18.4	33.0	13.5
**Peak height (AU/L)**						
Samples, *n*	23	43	25	49	14	22
Median (IQR)	203 (140-268)	289 (254-422)	309 (240-395)	325 (292-379)	193 (98-243)	349 (279-380)
Range	64-371	132- 544	206-507	158-511	8-354	168-499
CV, %	40.7	33.0	29.2	22.9	63.0	23.8
**ETP (AU⋅min/L)**						
Samples, *n*	23	43	25	49	14	22
Median (IQR)	1391 (1176-1774)	1832 (1322-2480)	1698 (1280-1961)	1711 (1436-1978)	1036 (724-1501)	1741 (1602-2290)
Range	601-2371	557-3572	971-3095	924-2958	80-2078	1111-2715
CV, %	30.5	38.3	34.1	25.5	56.1	24.9

AU, arbitrary unit; CV**,** coefficient of variation; ETP, endogenous thrombin potential; Tlag, lag time**;** Tmax, peak time; Tmin, trough;

We sought to identify covariates, namely DOAC plasma concentrations together with individual characteristics, which are potentially associated with Vi and Cmax_ATG at Tmin and Tmax (Supplementary Table 3). In multivariate analysis, at trough level (Tmin), apixaban and dabigatran concentrations were the only predictors of the interindividual variability of both Cmax_ATG and Vi. In patients on rivaroxaban at Tmin, cardiac failure was the only predictor of Cmax_ATG. At peak level, apixaban and dabigatran concentrations were predictors of the interindividual variability of Cmax_ATG; in patients on rivaroxaban, no predictors were found.

## 4 Discussion

In the present large-scale prospective study, we simultaneously evaluated fibrinography and thrombography using TD-4D system in plasma samples from very elderly patients receiving direct oral FXa or thrombin inhibitors for AF, along with spiked pooled normal plasma samples. For the first time, we demonstrated the ability of TD-4D system to assess the *in vitro* and *ex vivo* effects of DOACs, which reduced TG and subsequently delayed and slowed fibrin clot formation.

We first showed that rivaroxaban, apixaban, and dabigatran exerted a significant concentration-dependent effect on most thrombography and fibrinography parameters, both in spiked normal pooled plasma samples and in ADAGE patient samples. Thrombography results, ie, the prolongation of temporal parameters and the decrease in Cmax_ATG and ETP_ATG, were consistent with those obtained from other thrombography systems, such as calibrated automated thrombogram (CAT), using tissue-factor micelles in suspension [17,[19], [20], [21], [22], [23], [24], [25], [26], [27], [28], [29], [30], [31]]. However, unlike CAT, we did not observe a paradoxical increase in TG at low dabigatran concentrations, either in spiked or patient samples. Indeed, an apparent enhancement of TG has been observed with CAT system, most probably due to different calculation algorithms [44,[54], [55], [56]]. In contrast, TD-4D was able to assess both direct FXa and thrombin inhibitor effects on TG. Another advantage of the TD-4D analyzer is that it requires only 120 μL of plasma samples, making it particularly suitable for geriatric and pediatric patients. One disadvantage is that thrombin is quantified in arbitrary units, not in nanomolar, making comparisons with other thrombography systems difficult.

We further evidenced that DOACs affected fibrinography parameters in a concentration-dependent manner, increasing the lag time and decreasing the rates of growth at initiation and at 30 minutes (when measurable), as well as the CS. Moreover, we evidenced strong associations between thrombography and fibrinography parameters, especially for temporal parameters. Our results demonstrate that the delay in clot formation and decrease in clot growth reflect the decrease in TG induced by DOACs. In other words, the TD-4D system mimics the common coagulation pathway until final substrate (fibrinogen) transformation. Besides, we observed that D was found to be strongly correlated with fibrinogen concentration measured using the Clauss method, especially in ADAGE patients, most of whom had hyperfibrinogenemia. Interestingly, we found that the measured D was not sensitive to anti-FXa DOACs, regardless of the drug concentration. In contrast, it was sensitive to dabigatran concentration, probably due to its direct, reversible, competitive inhibitory effect on thrombin, whether free or bound to a fibrin clot, thus probably reflecting some analytical interference.

In addition, we evidenced substantial interindividual variability in thrombography and fibrinography parameters with increasing concentrations of DOACs in patients. We highlighted an underlying hypercoagulable state in some ADAGE patients, for whom shorter temporal parameters, combined with higher peak heights and endogenous thrombin potential, were observed compared with spiked pooled normal plasma. It is well known that advanced age and chronic comorbid conditions lead to an imbalance between procoagulant factors and natural coagulation inhibitors, potentially resulting in hypercoagulability [57]. This variability deserves further studies in a geriatric setting.

Besides the relationship between PD parameters and DOAC concentrations in ADAGE patients, we also assessed the interindividual variability of DOAC PK/PD in a subset of patients who were sampled at peak and/or trough levels. PK variability was high regardless of the DOAC; all CVs of plasma concentration at Tmax and Tmin were around ≥50%, as consistently reported by different groups, including ours, in patients in this age group [[9], [10], [11], [12],^14^,^15^,^17^]. As a reminder, ADAGE patients, with a mean age of 87 years and 69% being women, had substantial comorbid conditions (eg, renal impairment) and frequent polymedication, which contribute to the PK variability in this fragile population [17]. Using TD-4D, we evidenced that the variability of thrombography parameters, eg, peak height, was elevated in patients receiving rivaroxaban 15 mg o.d. and dabigatran 110 mg twice daily at Tmax (>40%), but less important in patients receiving 2.5 mg twice daily apixaban at both Tmax and Tmin (<30%). Finally, even though the sample size was limited, apixaban or dabigatran concentrations at Tmax and Tmin were the main contributors to Cmax_ATG and subsequently to the initial rate of growth, whereas cardiac failure in patients on rivaroxaban was the main determinant of Cmax_ATG at Tmin. These findings, obtained with TD-4D based on a coagulation process initiated with immobilized TF, remarkably confirm our previous results, which were obtained using another thrombography system (ST Genesia, Stago, soluble TF) for analyzing rivaroxaban and apixaban ADAGE samples [17], thus reinforcing the relevance of TD-4D in assessing DOAC PD. Although rivaroxaban has an impact on TD-4D parameters, rivaroxaban concentration was not found predictors when considering only concentrations at Tmin or Tmax. This is explained by the lower span of concentration ranges, the smaller sample size, and the high variability of parameters, all of which lower power.

Another interest of TD-4D was the evaluation of spatial propagation of thrombin and clot growth. However, an early Tsp (before 30 minutes) hampered the recording of some parameters (namely V and stationary amplitude of thrombin peak) in a substantial number of plasma samples, either from DOAC-free elderly subjects or ADAGE patients on DOACs. Some authors have proposed centrifuging samples at 10,000 × *g* to decrease Tsp [46,58], although this centrifugation condition is not recommended for performing TG tests. Lipets et al. [58] hypothesized that the combination of circulating active factors, circulating procoagulants, and contact pathway-activating extracellular vesicles is the predominant mechanism causing Tsp in patient plasma. Nevertheless, whether there is clinical relevance to Tsp or not needs to be further investigated [58,59].

Our study has several limitations. First, this study was conducted in a subset of ADAGE patients, depending on the availability of plasma samples, and not in the whole cohort. Moreover, the recruitment of patients with dabigatran was difficult, with fewer patients included than expected. Second, frequent early Tsp has limited the collection of the temporal-spatial parameters in a number of samples. Evaluating PD using TD-4D on fresh plasma or centrifuging plasma at high speed may solve this problem and overcome these drawbacks, yet this cannot be verified in ADAGE patients, as aliquots had already been prepared. Third, DOAC-free elderly individuals were not matched with ADAGE patient characteristics. A study including a larger number of matched controls (based on kidney/liver function) and a greater number of patients in the subgroups is of interest to better understand PD variability in the very elderly. Finally, the present study was not designed to evaluate the potential relationship between PK/PD data and clinical events, as it was underpowered to answer this question.

In conclusion, in this large-scale prospective study, fibrinography and thrombography simultaneously assessed with TD-4D provided consistent PD results for all 3 DOACs, including dabigatran. Substantial PD variability was observed. Apixaban and dabigatran concentrations at Tmin were the only significant predictors of interindividual variability in both Cmax_ATG (thrombography) and Vi (fibrinography). In rivaroxaban patients, cardiac failure was significantly associated with Cmax_ATG variability. The clinical relevance of such variability remains to be demonstrated.

## Supplementary material

Supplementary Videos

Supplementary Videos

Supplementary Videos
