# Supplementary material for: Fibrinography and thrombography (thrombodynamics-4D) in atrial fibrillation assessment of direct oral anticoagulants in geriatrics patients aged 80 years and older receiving direct oral anticoagulant therapy
Source: Res Pract Thromb Haemost. 2025 Jul 15;9(5):102969. doi: 10.1016/j.rpth.2025.102969 (PMC12351382; doi:10.1016/j.rpth.2025.102969)

Supplementary Figures

Supplementary Figure 1: Relationship between fibrinogen levels and fibrinography parameters in all ADAGE patients

(A) lag time (Tlag); (B) initial rate of clot growth (V); (C) clot size (CS) at 30 min; (D) clot density; (E) Time to spontaneous clotting (Tsp). ADAGE patients on rivaroxaban (red); on apixaban b.i.d. (blue); on dabigatran (green). Dashed lines indicate the locally determined reference intervals in healthy subjects.

Supplementary Figure 2: Fibrinography parameters as a fonction of thrombography parameters in ADAGE patients. I. TG lag time (Lag_ATG); II. TG time to peak (Tmax_ATG); III. TG peak-height (maximal thrombin concentration – Cmax ATG); IV. TG ETP_ATG; V. TGT stationnary amplitude of thrombin moving peak (Ast)

Supplement videos (3 files): videos showing the clot formation in an 85-year male patient on 15-mg rivaroxaban at three-time points after drug intake (namely, 3 hours - peak, 6 hours and 24h – trough level). The rivaroxaban concentrations were 197, 127 and 20 ng/mL, respectively. The process of fibrin clot formation was recorded in time-lapse video microscopy mode by means of a dark-field light scattering method.

Supplementary Figure 1: Relationship between fibrinogen level and fibrinography parameters in ADAGE patients

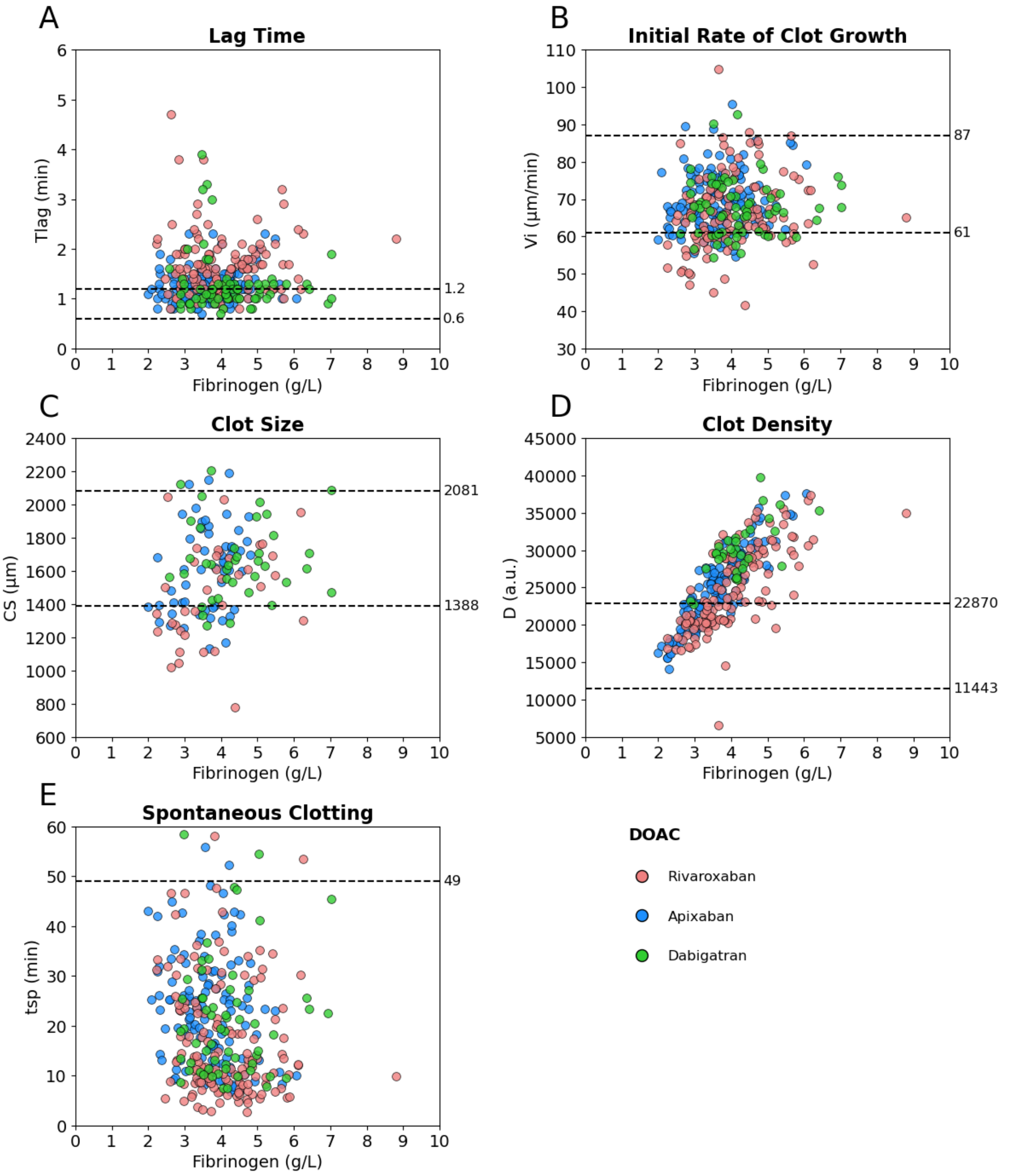

Supplementary Figure 2: Fibrinography parameters as a fonction of TG lag time and time to peak in ADAGE patients

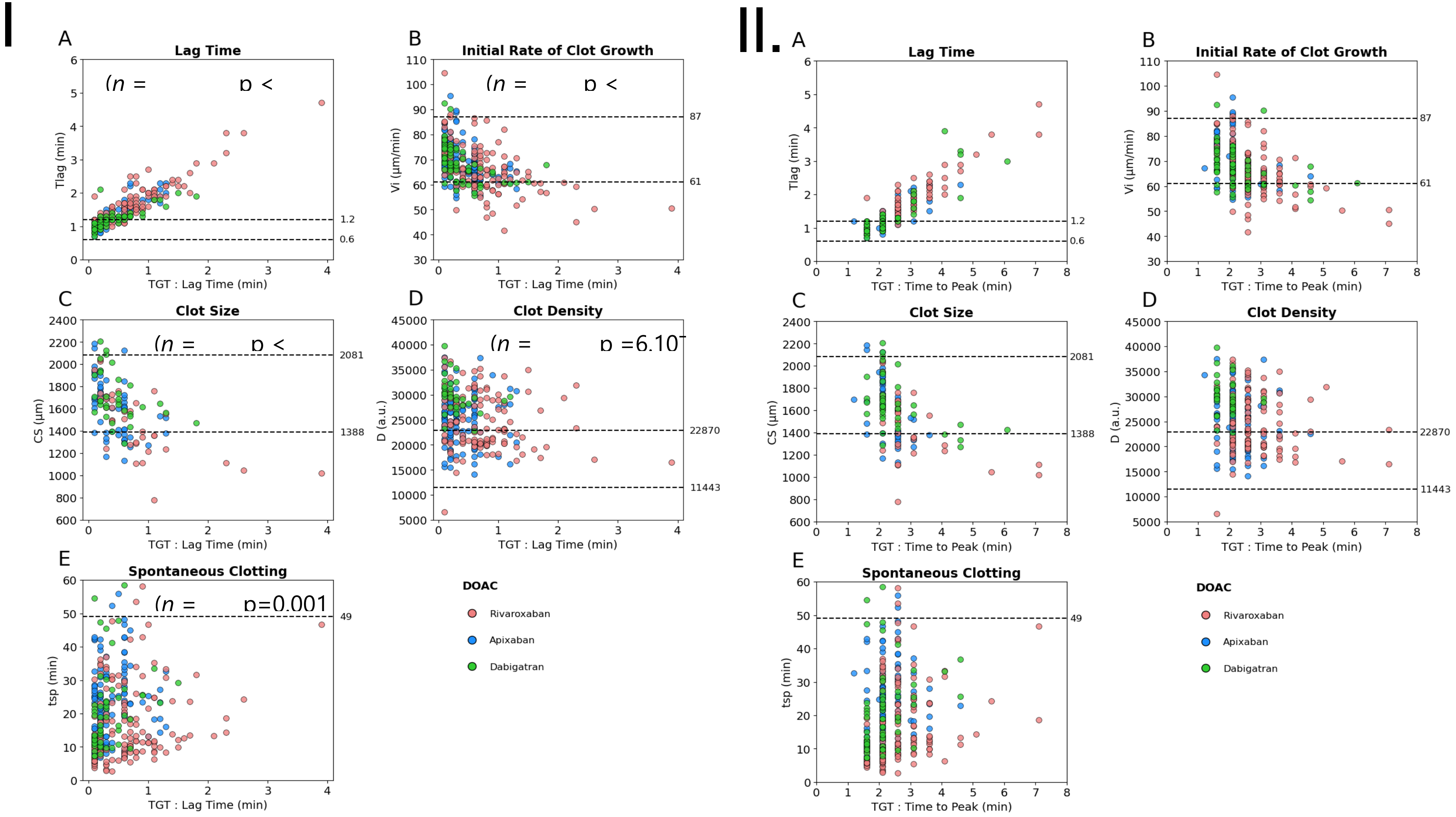

Supplementary Figure 2 (continued): Fibrinography parameters as a fonction of TG peak height and endogenous thrombin potential (ETP) in ADAGE patients

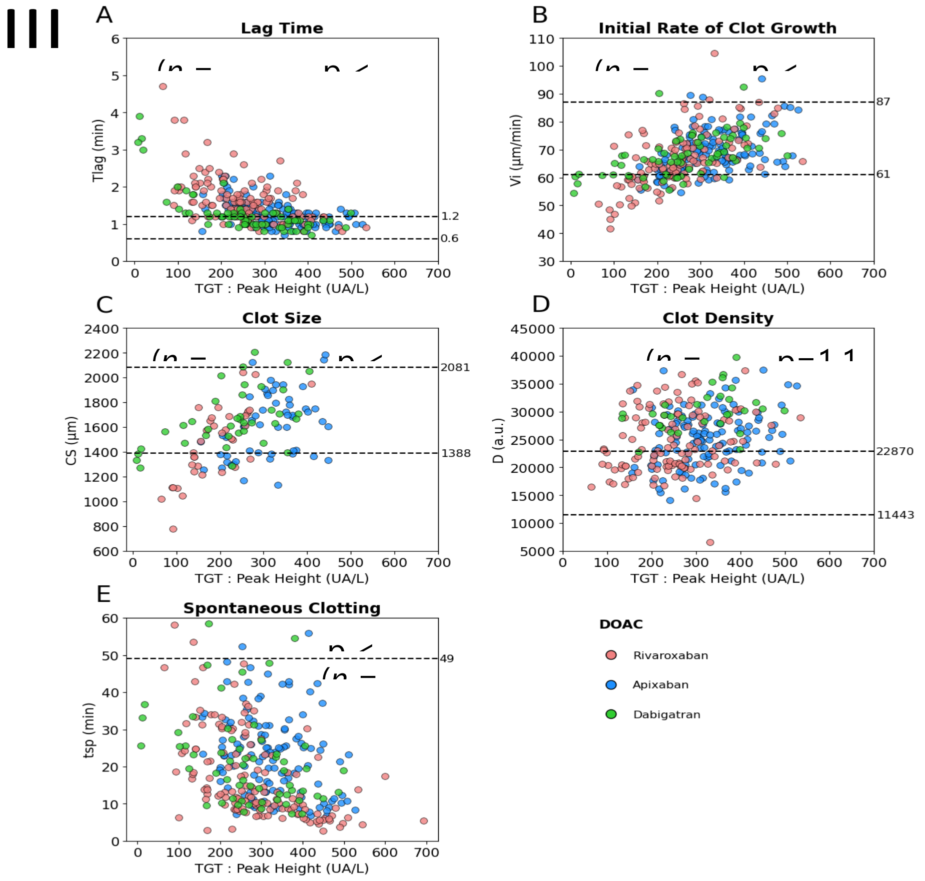

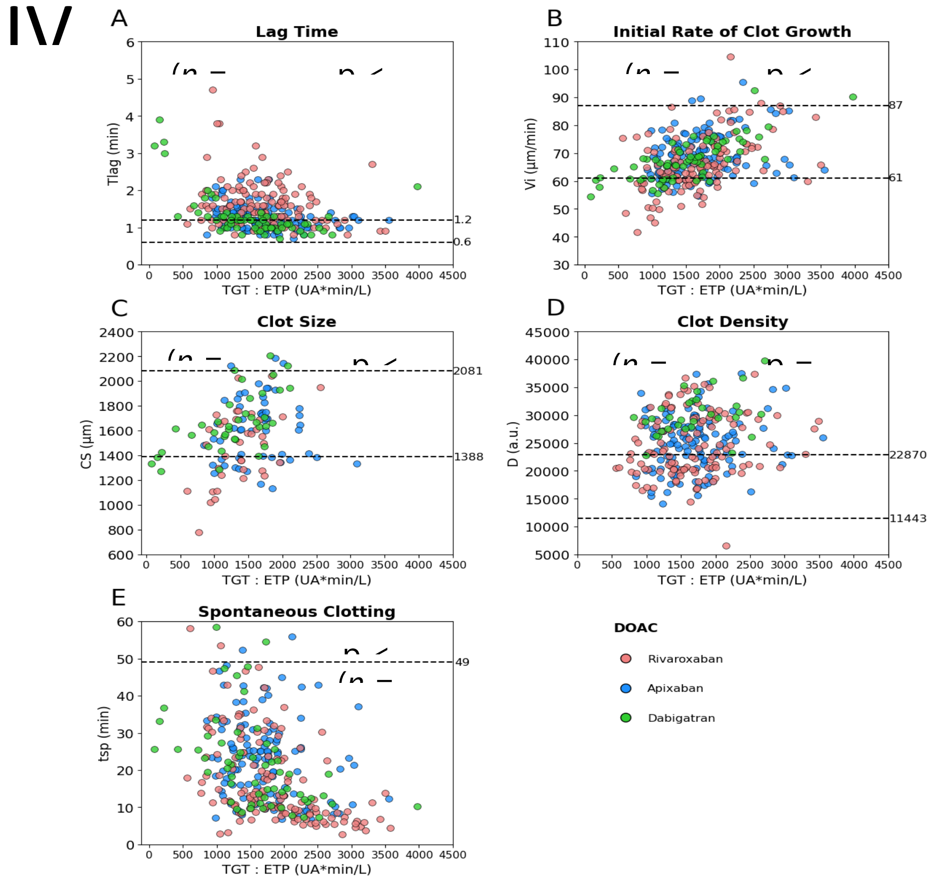

Supplementary Figure 2 (continued): Fibrinography parameters as a fonction of TG stationnary amplitude

V.

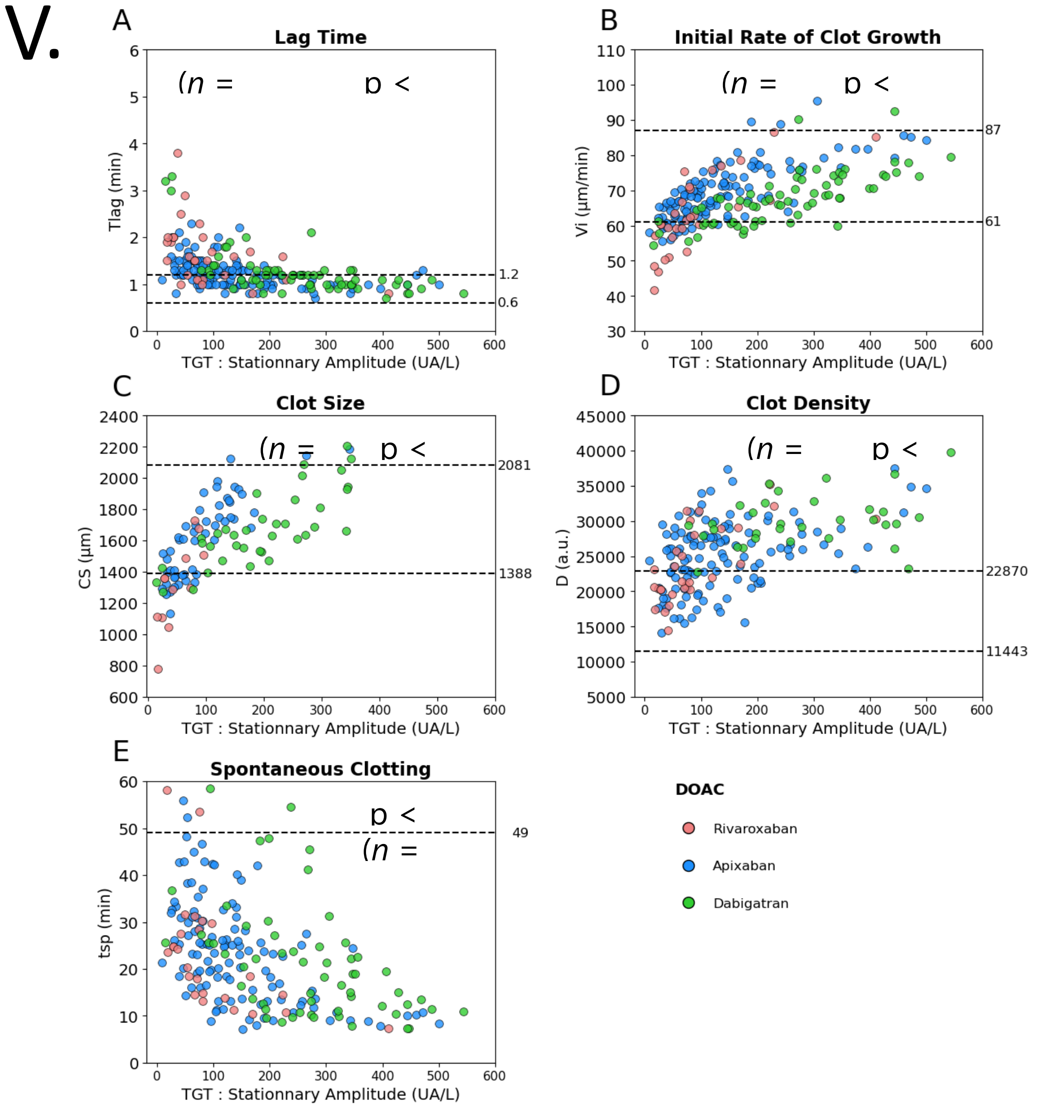

Supplement: Supplementary Figures [file mmc4.pdf]
