# Supplementary material for: Fibrinography and thrombography (thrombodynamics-4D) in atrial fibrillation assessment of direct oral anticoagulants in geriatrics patients aged 80 years and older receiving direct oral anticoagulant therapy
Source: Res Pract Thromb Haemost. 2025 Jul 15;9(5):102969. doi: 10.1016/j.rpth.2025.102969 (PMC12351382; doi:10.1016/j.rpth.2025.102969)
Supplement: Supplementary Tables [file mmc5.pdf]

Supplementary Tables

Supplementary Table 1.

TD-4D parameter (unit)	Definition
**Fibrinography**	
Lag time (Tlag, min)	Time from the start of measurement (contact of activator with plasma sample) until the onset of clot formation
Initial rate of clot growth (Vi, µm/min)	Mean clot growth velocity calculated on the 2-6 min interval after the beginning of clot growth
Rate of clot growth (V, µm/min)	Mean clot growth velocity on the interval 15-25 min after the beginning of clot growth or on the 10-min interval preceding spontaneous clot appearance
Clot size (CS, µm)	Clot size measured at 30 min
Clot density (D, a.u.)	Optical parameter corresponding to the intensity of light scattering from the fibrin clot
Time to spontaneous clotting (Tsp, min)	Time from the start of measurement until the average area of spontaneous clots (formed independently of the activator surface) reaches 5% of overall area of the measurement region.
**Thrombography parameter (unit)**	
Lag time (Lag_ATG, min)	Time when thrombin concentration reaches 20 AU/L
Time to thrombin peak (Tmax_ATG, min)	Time to maximal concentration of thrombin
Maximal thrombin concentration (*i.e.* thrombin peak-height) (Cmax_ATG, AU/L)	Maximal concentration of thrombin expressed in arbitrary units (AU)
Endogenous thrombin potential (ETP_ATG, AU•min/L)	Area under the curve of activator thrombin generation as a function of time
Stationary amplitude of moving peak of thrombin (Ast, AU/L)	Height of the peak calculated as a maximal activity of thrombin in the fibrin formation zone, which moves from the activator while clot grows
Rate of thrombin peak propagation (Vt, µm/min)	Spatial rate of thrombin propagation measured in the interval 45-55 min after the beginning of clot growth

Supplementary Table 2. Results of TD-4D parameters measured in pooled normal plasma and DOAC free elderly subjects

Parameter	**Pooled normal plasma Cryocheck (batch A1236)** ***n* = 18 runs** Mean ± SD (CV)	**DOAC free elderly subjects** ***n* = 35** Mean ± SD (CV)	**Locally determined reference intervals** (min-max)
**Fibrinography**			
Lag time (min)	0.8 ± 0.1 (7.1%)	0.9 ± 0.1 (15.6%)	[0.6 - 1.2]
Initial rate of Clot Growth (µm/min)	67.9 ± 1.5 (2.1%)	70.3 ± 6.0 (8.5%)	[61 – 87]
Clot size (µm)	1484 ± 30 (2.0%)	1669 ± 113 (6.8%)	[1388 – 2081]
Clot density (a.u)	16783 ± 454 (2.7%)	26108 ± 5346 (20.5%)	[11443 – 22870]
Time to spontaneous clotting (min)	36 ± 11 (29.7%)	14 ± 6 (43.4%)	21 ± 14 Mean ± SD
**Thrombography**			
ATG Lag time (min)	0.1*	0.1*	[0.1 - 0.2]
ATG Time to peak (min)	1.6*	1.7 ± 0.2 (11.3%)	[1.3 - 2.2]
ATG Maximum thrombin concentration (AU/L)	386 ± 21 (5.5%)	376 ± 96 (25.7%)	[157 – 453]
ETP- thrombin potential (AU•min/L)	1642 ± 63 (3.8%)	1780 ± 522 (29.3%)	[821 – 2386]
Stationary amplitude of thrombin peak (Ast) (AU/L)	42 ± 7 (16.7%)	246 ± 84 (34.0%)	ND

CV: coefficient of variation. AU: arbitrary unit (thrombinograhy); a.u.: arbitrary unit (fibrinobinograhy); ND: not determined - *CV for Lag time (thrombinography) and Time to peak could not be obtained due to the poor resolution of the system (1 measurement every 30 seconds). ND: not determined due to a too small number of available values

.

Supplementary Table 3 (see below)

Analysis for patients taking rivaroxaban

Thrombinography: peak height (PH)

Variable	Univariate analysis: T_{max} Effect	T_{max} %	T_{max} *p*	Univariate analysis: T_{min} Effect	T_{min} %	T_{min} *p*	Multivariate analysis: T_{max} Effect	T_{max} *p*	Multivariate analysis: T_{min} Effect	T_{min} *p*
**AOD Concentration**	$\beta_{C.pic}$ ≈ 0.1032 %	8.934 %	ANVA: 0.0797	$\beta_{C.res}$ ≈ 0.238 %	8.202 %	ANVA: 0.0270	*Not applicable*			
Halving	[−0.01307 ; 0.2195]		NP: 0.1288	[0.02819 ; 0.4482]		NP: 0.0382				
**Creatinin Clairance**	β_{ClCr} ≈ −11.05 %	22.77 %	ANVA: 0.0433	β_{ClCr} ≈ −0.8459 %	0.1696 %	ANVA: 0.7722	β_{ClCr} ≈ −8.927 % [−19.11 ; 2.538]	0.1171	β_{ClCr} ≈ −0.1807 % [−5.807 ; 5.781]	0.9503
10 units decrease	[−20.58 ; −0.38]		NP: 0.0847	[−6.494 ; 5.143]		NP: 0.8273	$\beta_{C.pic}$ ≈ −22.57 % [−48.77 ; 17.04]	C: 0.2144	$\beta_{C.res}$ ≈ −12.11 % [−24.29 ; 2.02]	C: 0.0881
**Heart Failure**	β_{Yes} ≈ −17.55 %	2.127 %	ANVA: 0.3159	β_{Yes} ≈ −26.04 %	18.74 %	ANVA: 0.0014	β_{Yes} ≈ −19.65 % [−44.54 ; 16.4]	0.2359	β_{Yes} ≈ −24.06 % [−37.16 ; −8.22]	0.0053
Reference: No	[−44.04 ; 21.47]		NP: 0.3698	[−38.16 ; −11.55]		NP: 0.0011	$\beta_{C.pic}$ ≈ −31.48 % [−54.09 ; 2.251]	C: 0.0631	$\beta_{C.res}$ ≈ −6.095 % [−18.67 ; 8.422]	C: 0.3834
**Amiodarone**	β_{Yes} ≈ −39.37 %	7.547 %	ANVA: 0.1025	β_{Yes} ≈ −20.81 %	5.311 %	ANVA: 0.0998	β_{Yes} ≈ −36.88 % [−64.94 ; 13.63]	0.1197	β_{Yes} ≈ −15.7 % [−37.16 ; 13.09]	0.2482
Reference: No	[−66.97 ; 11.29]		NP: 0.1297	[−40.12 ; 4.725]		NP: 0.0752	$\beta_{C.pic}$ ≈ −28.55 % [−51.75 ; 5.813]	C: 0.0902	$\beta_{C.res}$ ≈ −9.554 % [−22.48 ; 5.527]	C: 0.1967
**Other P-gp Inhibitor**	β_{Yes} ≈ −15.15 %	5.978 %	ANVA: 0.3900	β_{Yes} ≈ 13.12 %	1.489 %	ANVA: 0.2165	β_{Yes} ≈ −16.81 % [−42.52 ; 20.39]	0.3155	β_{Yes} ≈ 11.81 % [−7.971 ; 35.84]	0.2547
Reference: No	[−42.3 ; 24.79]		NP: 0.3291	[−7.186 ; 37.86]		NP: 0.2881	$\beta_{C.pic}$ ≈ −31.09 % [−53.95 ; 3.122]	C: 0.0687	$\beta_{C.res}$ ≈ −11.65 % [−23.66 ; 2.259]	C: 0.0950
**Other CYP Inhibitor**	β_{Yes} ≈ −14.51 %	3.162 %	ANVA: 0.4239	β_{Yes} ≈ −18.31 %	9.023 %	ANVA: 0.0414	β_{Yes} ≈ −17.35 % [−43.47 ; 20.84]	0.3120	β_{Yes} ≈ −14.5 % [−30.98 ; 5.903]	0.1476
Reference: No	[−42.46 ; 27.04]		NP: 0.3628	[−32.72 ; −0.8155]		NP: 0.0240	$\beta_{C.pic}$ ≈ −31.6 % [−54.34 ; 2.452]	C: 0.0643	$\beta_{C.res}$ ≈ −7.741 % [−21.34 ; 8.203]	C: 0.3146

Fibrinography: Initial Velocity of Clot Formation

Variable	Univariate analysis: T_{max} Effect	T_{max} %	T_{max} *p*	Univariate analysis: T_{min} Effect	T_{min} %	T_{min} *p*	Multivariate analysis: T_{max} Effect	T_{max} *p*	Multivariate analysis: T_{min} Effect	T_{min} *p*
**AOD Concentration**	$\beta_{C.pic}$ ≈ −0.001769 %	0.1867 %	ANVA: 0.9271	$\beta_{C.res}$ ≈ 0.08094 %	11.58 %	ANVA: 0.0409	*Not applicable*			
Halving	[−0.04115 ; 0.03763]		NP: 0.9647	[0.003548 ; 0.1584]		NP: 0.0090				
**Creatinin Clairance**	β_{ClCr} ≈ −2.939 %	7.164 %	ANVA: 0.1653	β_{ClCr} ≈ −1.974 %	9.841 %	ANVA: 0.0519	β_{ClCr} ≈ −2.291 % [−6.121 ; 1.696]	0.2428	β_{ClCr} ≈ −1.935 % [−3.686 ; −0.1524]	0.0344
10 units decrease	[−7.019 ; 1.32]		NP: 0.1024	[−3.925 ; 0.01701]		NP: 0.1323	$\beta_{C.pic}$ ≈ −1.369 % [−12.84 ; 11.61]	C: 0.8196	$\beta_{C.res}$ ≈ −6.026 % [−10.72 ; −1.086]	C: 0.0189
**Fibrinogenemia**	β_{Fb} ≈ 7.663 %	26.48 %	ANVA: 0.0059	β_{Fb} ≈ 3.309 %	8.754 %	ANVA: 0.0637	*Not applicable*			
1 unit increase	[2.361 ; 13.24]		NP: 0.0041	[−0.195 ; 6.935]		NP: 0.0465				
**Heart Failure**	β_{Yes} ≈ −7.731 %	7.593 %	ANVA: 0.1860	β_{Yes} ≈ −1.504 %	0.3981 %	ANVA: 0.7068	β_{Yes} ≈ −6.304 % [−15.89 ; 4.381]	0.2248	β_{Yes} ≈ −0.992 % [−8.439 ; 7.061]	0.7969
Reference: No	[−18.31 ; 4.216]		NP: 0.1500	[−9.172 ; 6.812]		NP: 0.7538	$\beta_{C.pic}$ ≈ −3.778 % [−14.44 ; 8.217]	C: 0.5045	$\beta_{C.res}$ ≈ −6.291 % [−11.47 ; −0.812]	C: 0.0263
**Amiodarone**	β_{Yes} ≈ −14.24 %	9.25 %	ANVA: 0.1119	β_{Yes} ≈ −14.93 %	22.91 %	ANVA: 0.0013	β_{Yes} ≈ −7.146 % [−23.23 ; 12.31]	0.4284	β_{Yes} ≈ −10.29 % [−19.36 ; −0.1991]	0.0461
Reference: No	[−29.21 ; 3.899]		NP: 0.0875	[−22.57 ; −6.526]		NP: 0.0015	$\beta_{C.pic}$ ≈ −2.8 % [−13.89 ; 9.711]	C: 0.6321	$\beta_{C.res}$ ≈ −4.314 % [−9.536 ; 1.209]	C: 0.1194
**Other P-gp Inhibitor**	β_{Yes} ≈ −4.947 %	3.367 %	ANVA: 0.4092	β_{Yes} ≈ 8.943 %	12.45 %	ANVA: 0.0280	β_{Yes} ≈ −5.834 % [−15.72 ; 5.21]	0.2737	β_{Yes} ≈ 8.227 % [0.7211 ; 16.29]	0.0321
Reference: No	[−16.06 ; 7.637]		NP: 0.4717	[0.9826 ; 17.53]		NP: 0.0261	$\beta_{C.pic}$ ≈ −4.052 % [−14.78 ; 8.026]	C: 0.4777	$\beta_{C.res}$ ≈ −5.2 % [−10.04 ; −0.09747]	C: 0.0461
**Other CYP Inhibitor**	β_{Yes} ≈ −2.699 %	1.545 %	ANVA: 0.6680	βYes ≈ −7.457 %	9.905 %	ANVA: 0.0514	β_{Yes} ≈ −1.191 % [−11.99 ; 10.93]	0.8323	β_{Yes} ≈ −6.013 % [−13.23 ; 1.803]	0.1238
Reference: No	[−14.53 ; 10.77]		NP: 0.8667	[−14.4 ; 0.05099]		NP: 0.0694	$\beta_{C.pic}$ ≈ −3.674 % [−14.76 ; 8.856]	C: 0.5328	$\beta_{C.res}$ ≈ −4.69 % [−10.07 ; 1.011]	C: 0.1020

Legend

C.pic : DOAC Concentration at T_{max} (peak)
C.res : DOAC Concentration at T_{min} (trough)
Fb : Fibrinogen plasma concentration
ClCr : Creatinin Clearance, according to the Cockroft & Gault formula
ANVA : Analysis of variance
NP : Non-parametric (Wilcoxon-Mann-Whitney or Spearman's rank correlation coefficient)

Not applicable — *Multivariate analysis cannot be performed for these variables (see Methods for details)*

Analysis for patients taking apixaban

Thrombinography: peak height (PH)

Variable	Univariate analysis						Multivariate analysis			
	T_{max}			T_{min}			T_{max}		T_{min}	
	Effect	%	*p*	Effect	%	*p*	Effect	*p*	Effect	*p*
**AOD Concentration**	$\beta_{C.pic} \approx 0.07471$ %	16.66 %	ANVA: 0.0177	$\beta_{C.res} \approx 0.1698$ %	30.28 %	ANVA: < 0.0001	*Not applicable*			
Halving	[0.01399 ; 0.1355]		NP: 0.0041	[0.1061 ; 0.2336]		NP: < 0.0001				
**Creatinin Clairance**	$\beta_{ClCr} \approx 1.044$ %	1.117 %	ANVA: 0.6943	$\beta_{ClCr} \approx 0.2694$ %	0.2372 %	ANVA: 0.8971	$\beta_{ClCr} \approx 1.716$ % [−3.073 ; 6.742]	0.4753	$\beta_{ClCr} \approx 0.9457$ % [−2.577 ; 4.596]	0.5976
10 units decrease	[−4.224 ; 6.601]		NP: 0.9693	[−3.804 ; 4.516]		NP: 0.9128	$\beta_{C.pic} \approx -23.52$ % [−37.05 ; −7.073]	C: 0.0088	βC.res ≈ −19.24 % [−26.25 ; −11.58]	C: < 0.0001
**Heart Failure**	$\beta_{Yes} \approx 3.12$ %	0.8026 %	ANVA: 0.7747	$\beta_{Yes} \approx -2.235$ %	0.05363 %	ANVA: 0.7166	$\beta_{Yes} \approx 8.211$ % [−11.17 ; 31.83]	0.4192	$\beta_{Yes} \approx -1.673$ % [−11.58 ; 9.346]	0.7515
Reference: No	[−17.06 ; 28.21]		NP: 0.8513	[−13.64 ; 10.68]		NP: 0.8319	$\beta_{C.pic} \approx -24.04$ % [−37.57 ; −7.564]	C: 0.0078	$\beta_{C.res} \approx -19.06$ % [−26.07 ; −11.39]	C: < 0.0001
**Amiodarone**	$\beta_{Yes} \approx 5.428$ %	0.8145 %	ANVA: 0.6563	$\beta_{Yes} \approx 0.685$ %	0.0008444 %	ANVA: 0.9276	$\beta_{Yes} \approx 14.88$ % [−7.726 ; 43.03]	0.2049	$\beta_{Yes} \approx 5.127$ % [−7.577 ; 19.58]	0.4401
Reference: No	[−17.13 ; 34.12]		NP: 0.6622	[−13.32 ; 16.96]		NP: 0.8406	$\beta_{C.pic} \approx -25.46$ % [−38.77 ; −9.263]	C: 0.0049	$\beta_{C.res} \approx -19.49$ % [−26.5 ; −11.81]	C: < 0.0001
**Other P-gp Inhibitor**	$\beta_{Yes} \approx 10.78$ %	1.718 %	ANVA: 0.4661	$\beta_{Yes} \approx -5.264$ %	0.6428 %	ANVA: 0.4837	$\beta_{Yes} \approx 1.261$ % [−22.55 ; 32.39]	0.9243	$\beta_{Yes} \approx -2.693$ % [−14.75 ; 11.07]	0.681
Reference: No	[−16.6 ; 47.16]		NP: 0.4812	[−18.75 ; 10.47]		NP: 0.6378	$\beta_{C.pic} \approx -22.8$ % [−36.97 ; −5.447]	C: 0.0143	$\beta_{C.res} \approx -18.95$ % [−25.99 ; −11.25]	C: < 0.0001
**Other CYP Inhibitor**	$\beta_{Yes} \approx -12.87$ %	4.729 %	ANVA: 0.2108	$\beta_{Yes} \approx -6.27$ %	2.346 %	ANVA: 0.3445	$\beta_{Yes} \approx -6.009$ % [−24.03 ; 16.28]	0.5551	$\beta_{Yes} \approx -2.328$ % [−13.25 ; 9.964]	0.6921
Reference: No	[−30.09 ; 8.607]		NP: 0.1686	[−18.19 ; 7.388]		NP: 0.4414	$\beta_{C.pic} \approx -21.62$ % [−36.06 ; −3.923]	C: 0.0208	$\beta_{C.res} \approx -18.86$ % [−25.96 ; −11.08]	C: < 0.0001

Fibrinography: Initial Velocity of Clot Formation

Variable	Univariate analysis						Multivariate analysis			
	T_{max}			T_{min}			T_{max}		T_{min}	
	Effect	%	*p*	Effect	%	*p*	Effect	*p*	Effect	*p*
**AOD Concentration**	$\beta_{C.pic} \approx 0.02018$ %	7.18 %	ANVA: 0.1755	$\beta_{C.res} \approx 0.04558$ %	12.05 %	ANVA: 0.0063	*Not applicable*			
Halving	[−0.009578 ; 0.04994]		NP: 0.1827	[0.01343 ; 0.07774]		NP: 0.0033				
**Creatinin Clairance**	$\beta_{ClCr} \approx -0.136$ %	0.005309 %	ANVA: 0.9119	$\beta_{ClCr} \approx -0.3293$ %	0.1701 %	ANVA: 0.7161	$\beta_{ClCr} \approx 0.2425$ % [−2.086 ; 2.627]	0.8337	$\beta_{ClCr} \approx -0.1016$ % [−1.794 ; 1.62]	0.9055
10 units decrease	[−2.603 ; 2.394]		NP: 0.6572	[−2.116 ; 1.49]		NP: 0.4111	$\beta_{C.pic} \approx -7.037$ % [−15.28 ; 2.011]	C: 0.1182	$\beta_{C.res} \approx -5.259$ % [−9.395 ; −0.9355]	C: 0.0186
**Fibrinogenemia**	$\beta_{Fb} \approx 5.016$ %	11.85 %	ANVA: 0.0647	$\beta_{Fb} \approx 2.872$ %	5.531 %	ANVA: 0.0772	*Not applicable*			
1 unit increase	[−0.3201 ; 10.64]		NP: 0.1704	[−0.3191 ; 6.166]		NP: 0.0591				
**Heart Failure**	$\beta_{Yes} \approx -2.711$ %	0.7177 %	ANVA: 0.5777	$\beta_{Yes} \approx -0.2992$ %	0.02997 %	ANVA: 0.9138	$\beta_{Yes} \approx -2.815$ % [−11.7 ; 6.961]	0.5450	$\beta_{Yes} \approx -1.191$ % [−6.295 ; 4.19]	0.6522
Reference: No	[−11.98 ; 7.528]		NP: 0.3245	[−5.654 ; 5.36]		NP: 0.9109	$\beta_{C.pic} \approx -6.44$ % [−14.78 ; 2.715]	C: 0.1544	$\beta_{C.res} \approx -5.253$ % [−9.37 ; −0.9497]	C: 0.0182
**Amiodarone**	$\beta_{Yes} \approx 3.158$ %	1.313 %	ANVA: 0.5676	$\beta_{Yes} \approx -4.571$ %	3.632 %	ANVA: 0.1637	$\beta_{Yes} \approx 5.668$ % [−4.813 ; 17.3]	0.2874	$\beta_{Yes} \approx -2.915$ % [−8.952 ; 3.522]	0.3596
Reference: No	[−7.609 ; 15.18]		NP: 0.5178	[−10.7 ; 1.983]		NP: 0.0981	$\beta_{C.pic} \approx -8.106$ % [−16.3 ; 0.8877]	C: 0.0740	$\beta_{C.res} \approx -4.97$ % [−9.124 ; −0.6255]	C: 0.0262
**Other P-gp Inhibitor**	$\beta_{Yes} \approx 1.205$ %	0.1458 %	ANVA: 0.8528	$\beta_{Yes} \approx -2.537$ %	1.478 %	ANVA: 0.4471	$\beta_{Yes} \approx -0.3237$ % [−12.22 ; 13.18]	0.9585	$\beta_{Yes} \approx -1.248$ % [−7.397 ; 5.31]	0.6968
Reference: No	[−11.24 ; 15.39]		NP: 0.9777	[−8.877 ; 4.244]		NP: 0.4222	$\beta_{C.pic} \approx -6.993$ % [−15.48 ; 2.341]	C: 0.1310	$\beta_{C.res} \approx -5.228$ % [−9.354 ; −0.9139]	C: 0.0190
**Other CYP Inhibitor**	$\beta_{Yes} \approx -2.172$ %	0.5975 %	ANVA: 0.6681	$\beta_{Yes} \approx 2.974$ %	1.489 %	ANVA: 0.3297	$\beta_{Yes} \approx 2.283$ % [−7.819 ; 13.49]	0.6587	$\beta_{Yes} \approx 4.205$ % [−1.478 ; 10.22]	0.1466
Reference: No	[−11.83 ; 8.543]		NP: 0.7015	[−2.997 ; 9.313]		NP: 0.4325	$\beta_{C.pic} \approx -7.546$ % [−16.08 ; 1.858]	C: 0.1078	$\beta_{C.res} \approx -5.71$ % [−9.773 ; −1.465]	C: 0.0098

Legend

C.pic : DOAC Concentration at T_{max} (peak)

C.res : DOAC Concentration at T_{min} (trough)

Fb : Fibrinogen plasma concentration

ClCr : Creatinin Clearance, according to the Cockroft & Gault formula

ANVA : Analysis of variance

NP : Non-parametric (Wilcoxon-Mann-Whitney or Spearman's rank correlation coefficient)

Not applicable — *Multivariate analysis cannot be performed for these variables (see Methods for details)*

Analysis for patients taking dabigatran

Thrombinography: peak height (PH)

Variable	Univariate analysis						Multivariate analysis			
	T_{max}			T_{min}			T_{max}		T_{min}	
	Effect	%	*p*	Effect	%	*p*	Effect	*p*	Effect	*p*
**AOD Concentration**	$\beta_{C.pic}$ ≈ 0.4838 %	72.17 %	ANVA: < 0.0001	$\beta_{C.res}$ ≈ 0.4447 %	31.86 %	ANVA: 0.0002	*Not applicable*			
Halving	[0.3571 ; 0.6105]		NP: < 0.0001	[0.2311 ; 0.6588]		NP: 0.0118				
**Creatinin Clairance**	β_{ClCr} ≈ −19.13 %	21.05 %	ANVA: 0.3965	β_{ClCr} ≈ −4.31 %	10.31 %	ANVA: 0.1374	β_{ClCr} ≈ 13.33 % [−22.08 ; 64.84]	0.4739	β_{ClCr} ≈ −0.001213 % [−5.002 ; 5.263]	0.9996
10 units decrease	[−52.38 ; 37.34]		NP: 0.1896	[−9.82 ; 1.537]		NP: 0.1473	$\beta_{C.pic}$ ≈ −82.31 % [−92.76 ; −56.82]	C: 0.0015	$\beta_{C.res}$ ≈ −29.83 % [−41.81 ; −15.37]	C: 0.0008
**Heart Failure**	β_{Yes} ≈ −58.7 %	6.662 %	ANVA: 0.2355	β_{Yes} ≈ 2.901 %	0.0008228 %	ANVA: 0.8352	β_{Yes} ≈ −33.87 % [−71.42 ; 53.03]	0.3116	β_{Yes} ≈ 8.37 % [−12.08 ; 33.58]	0.4347
Reference: No	[−90.94 ; 88.28]		NP: 0.3034	[−22.27 ; 36.22]		NP: 0.9783	$\beta_{C.pic}$ ≈ −79.36 % [−87.68 ; −65.44]	C: p < 0.0001	$\beta_{C.res}$ ≈ −28.72 % [−38.84 ; −16.93]	C: 0.0001
**Amiodarone**	β_{Yes} ≈ −36.93 %	16.72 %	ANVA: 0.4494	β_{Yes} ≈ 9.51 %	0.1295 %	ANVA: 0.7829	β_{Yes} ≈ 52.2 % [−31.43 ; 237.8]	0.2756	β_{Yes} ≈ −14.27 % [−48.72 ; 43.29]	0.5412
Reference: No	[−82.3 ; 124.7]		NP: 0.0559	[−44.12 ; 114.6]		NP: 1.0000	$\beta_{C.pic}$ ≈ −82.11 % [−90.81 ; −65.16]	C: p < 0.0001	$\beta_{C.res}$ ≈ −28.98 % [−39.28 ; −16.94]	C: 0.0002
**Other P-gp Inhibitor**	β_{Yes} ≈ 272.6 %	41.61 %	ANVA: 0.0183	β_{Yes} ≈ −16.56 %	5.95 %	ANVA: 0.2218	β_{Yes} ≈ 66.41 % [−26.31 ; 275.8]	0.1999	β_{Yes} ≈ −11.33 % [−29.32 ; 11.23]	0.2838
Reference: No	[29.58 ; 971.1]		NP: 0.0160	[−38.06 ; 12.4]		NP: 0.2425	$\beta_{C.pic}$ ≈ −74.33 % [−87 ; −49.3]	C: 0.0008	$\beta_{C.res}$ ≈ −27.52 % [−37.71 ; −15.66]	C: 0.0002
**Other CYP Inhibitor**	β_{Yes} ≈ 29.33 %	0.2793 %	ANVA: 0.6884	β_{Yes} ≈ −4.848 %	0.4757 %	ANVA: 0.7090	β_{Yes} ≈ 144.9 % [31.01 ; 357.7]	0.0086	β_{Yes} ≈ −0.9858 % [−19.35 ; 21.56]	0.9213
Reference: No	[−66.38 ; 397.5]		NP: 0.6612	[−27.48 ; 24.85]		NP: 0.9159	$\beta_{C.pic}$ ≈ −83.08 % [−89.74 ; −72.09]	C: p < 0.0001	$\beta_{C.res}$ ≈ −28.2 % [−38.5 ; −16.18]	C: 0.0002

Fibrinography: Initial Velocity of Clot Formation

Variable	Univariate analysis						Multivariate analysis			
	T_{max}			T_{min}			T_{max}		T_{min}	
	Effect	%	*p*	Effect	%	*p*	Effect	*p*	Effect	*p*
**AOD Concentration**	$\beta_{C.pic}$ ≈ 0.01723 %	6.92 %	ANVA: 0.2371	$\beta_{C.res}$ ≈ 0.1281 %	37.46 %	ANVA: 0.0005	*Not applicable*			
Halving	[−0.01244 ; 0.04691]		NP: 0.1270	[0.06198 ; 0.1943]		NP: < 0.0001				
**Creatinin Clairance**	β_{ClCr} ≈ −0.4954 %	0.01478 %	ANVA: 0.8797	β_{ClCr} ≈ −1.072 %	5.531 %	ANVA: 0.2432	β_{ClCr} ≈ 3.466 % [−6.29 ; 14.24]	0.4564	β_{ClCr} ≈ 0.7499 % [−1.117 ; 2.652]	0.4135
10 units decrease	[−7.275 ; 6.78]		NP: 0.5583	[−2.903 ; 0.7921]		NP: 0.1150	$\beta_{C.pic}$ ≈ −13.3 % [−30.12 ; 7.58]	C: 0.1689	$\beta_{C.res}$ ≈ −12.33 % [−17.86 ; −6.434]	C: 0.0005
**Fibrinogenemia**	β_{Fb} ≈ −0.9758 %	0.4702 %	ANVA: 0.8309	β_{Fb} ≈ 1.175 %	0.9507 %	ANVA: 0.6296	*Not applicable*			
1 unit increase	[−9.985 ; 8.936]		NP: 0.8691	[−3.69 ; 6.286]		NP: 0.7947				
**Heart Failure**	β_{Yes} ≈ −10.67 %	9.689 %	ANVA: 0.1723	β_{Yes} ≈ −7.28 %	12.46 %	ANVA: 0.0699	β_{Yes} ≈ −8.813 % [−23.1 ; 8.128]	0.2666	β_{Yes} ≈ −5.922 % [−11.64 ; 0.1656]	0.0558
Reference: No	[−24.41 ; 5.573]		NP: 0.1383	[−14.6 ; 0.6678]		NP: 0.0753	$\beta_{C.pic}$ ≈ −7.358 % [−17.04 ; 3.452]	C: 0.1606	$\beta_{C.res}$ ≈ −9.586 % [−13.69 ; −5.283]	C: 0.0002
**Amiodarone**	β_{Yes} ≈ −8.222 %	10.47 %	ANVA: 0.2421	β_{Yes} ≈ 8.021 %	2.117 %	ANVA: 0.4538	β_{Yes} ≈ −5.463 % [−20.88 ; 12.95]	0.5047	β_{Yes} ≈ 2.153 % [−18.04 ; 27.33]	0.8429
Reference: No	[−21.06 ; 6.702]		NP: 0.3676	[−12.36 ; 33.15]		NP: 0.2569	$\beta_{C.pic}$ ≈ −5.6 % [−18.97 ; 9.975]	C: 0.4270	$\beta_{C.res}$ ≈ −9.929 % [−14.39 ; −5.237]	C: 0.0003
**Other P-gp Inhibitor**	β_{Yes} ≈ 6.959 %	5.973 %	ANVA: 0.3638	β_{Yes} ≈ −3.323 %	1.595 %	ANVA: 0.4725	β_{Yes} ≈ 8.254 % [−11.96 ; 33.1]	0.4193	β_{Yes} ≈ −1.375 % [−8.478 ; 6.28]	0.7047
Reference: No	[−8.281 ; 24.73]		NP: 0.4278	[−12.13 ; 6.371]		NP: 0.3774	$\beta_{C.pic}$ ≈ −5.1 % [−18.52 ; 10.53]	C: 0.4687	$\beta_{C.res}$ ≈ −9.87 % [−14.33 ; −5.182]	C: 0.0003
**Other CYP Inhibitor**	β_{Yes} ≈ 4.299 %	1.819 %	ANVA: 0.5902	β_{Yes} ≈ −4.13 %	4.801 %	ANVA: 0.3086	β_{Yes} ≈ 7.038 % [−10.01 ; 27.32]	0.4098	β_{Yes} ≈ −2.918 % [−9.013 ; 3.586]	0.3539
Reference: No	[−11.46 ; 22.86]		NP: 0.5833	[−11.83 ; 4.238]		NP: 0.3883	$\beta_{C.pic}$ ≈ −8.898 % [−20.85 ; 4.856]	C: 0.1743	$\beta_{C.res}$ ≈ −9.775 % [−14.14 ; −5.183]	C: 0.0003

Legend

C.pic : DOAC Concentration at T_{max} (peak)

C.res : DOAC Concentration at T_{min} (trough)

Fb : Fibrinogen plasma concentration

ClCr : Creatinin Clearance, according to the Cockroft & Gault formula

ANVA : Analysis of variance

NP : Non-parametric (Wilcoxon-Mann-Whitney or Spearman's rank correlation coefficient)

Not applicable — *Multivariate analysis cannot be performed for these variables (see Methods for details)*
